# The RBM39 degrader indisulam inhibits acute megakaryoblastic leukemia by altering the alternative splicing of ZMYND8

**DOI:** 10.1186/s13578-025-01380-3

**Published:** 2025-04-13

**Authors:** Ying Yang, Zhiheng Li, Yang Yang, Peifang Xiao, Zhixu He, Zimu Zhang, Yizhen Li, Lei Shi, Xiaodong Wang, Yanfang Tao, Junjie Fan, Fenli Zhang, Chunxia Yang, Fahua Yao, Tongting Ji, Yongping Zhang, Bi Zhou, Juanjuan Yu, Ailian Guo, Zhongling Wei, Wanyan Jiao, Yumeng Wu, Yan Li, Di Wu, Yijun Wu, Li Gao, Yixin Hu, Jian Pan, Shaoyan Hu, Xiaoyan Yang

**Affiliations:** 1 Department of Pediatrics, Affiliated Hospital of Guizhou Medical University, No. 28 Guiyi Street, Guiyang, 550001 China; 2 Institute of Pediatric Research, Children’s Hospital of Soochow University, No. 92 Zhongnan Street, SIP, Suzhou, 215003 China; 3 Department of Hematology, Children’s Hospital of Soochow University, No. 92 Zhongnan Street, SIP, Suzhou, 215003 China; 4 Department of Medicinal Chemistry, Jiangsu Key Laboratory of Drug Design and Optimization, China Pharmaceutical University, Nanjing, 210009 China; 5 Department of Orthopaedics, Children’s Hospital of Soochow University, Suzhou, 215003 China; 6 Department of Pediatrics, Guizhou Hospital, Shanghai Children’s Medical Center, Guiyang, 550004 China; 7 Children’s Hospital of Soochow University, Suzhou, 215003 China; 8 Department of Pediatrics, Suzhou Hospital Affiliated to Anhui Medical University, Suzhou Municipal Hospital of Anhui Province, Suzhou, 234000 China; 9 Department of Pediatric, Yancheng Third People’s Hospital, Yancheng, 224000 China; 10 Department of Pediatric, The First Affiliated Hospital of Bengbu Medical University, Bengbu, 233004 China; 11 Department of Pediatric, The Affiliated Hospital of Xuzhou Medical University, Xuzhou, 221000 China; 12 Jiangsu Pediatric Hematology and Oncology Center, Suzhou, 215003 China

**Keywords:** Acute megakaryoblastic leukemia, Indisulam, RBM39, Alternative splicing, ZMYND8

## Abstract

**Background:**

Acute megakaryoblastic leukemia (AMKL) is a rare hematological malignancy in adults but children. Alternative splicing (AS) has been shown to affect hematological cancer progression, making splicing factors promising targets. Our research aims to investigate the efficacy of the molecular glue degrader indisulam, which targets the splicing factor RNA binding motif protein 39 (RBM39) in AMKL models.

**Results:**

Public drug sensitivity data analysis revealed that AMKL cell lines exhibited the highest sensitivity to indisulam compared with other tumor types. Then we confirmed that RBM39 depletion by indisulam treatment induced AMKL cell cycle arrest and apoptosis. In AMKL mouse model, indisulam treatment significantly reduced the leukemic burden and prolonged the lifetime of AMKL mice. Mechanically, integration of transcriptomic and proteomic analyses revealed that indisulam-mediated RBM39 degradation resulted in AS of the transcription factor zinc finger MYND-type containing 8 (ZMYND8), an AMKL cell growth regulator. Finally, the effectiveness of indisulam depended on DDB1- and Cul4- Associated Factor 15 (DCAF15) expression because knockout of DCAF15 rescued the indisulam-induced RBM39 degradation and mis-splicing of ZMYND8.

**Conclusion:**

Indisulam is a promising therapeutic candidate for AMKL and the RBM39-mediated ZMYND8 splicing plays an important role in promoting the development of AMKL.

**Supplementary Information:**

The online version contains supplementary material available at 10.1186/s13578-025-01380-3.

## Background

Acute megakaryocytic leukemia (AMKL) is a heterogeneous subtype of acute myeloid leukemia (AML) based on the French American British (FAB) classification (AML M7) [1]. AMKL accounts for 4 to 15% of pediatric AML cases [2], with a significantly lower 5-year survival rate than other AML subtypes [3, 4]. Despite advances in treatment including intensive treatment regimens and allogeneic hematopoietic stem cell transplantation, there are still no individualized treatment approaches for AMKL which calls for the identification of novel treatment strategies [5].

Splicing factors cause splicing regulatory changes and errors closely associated with diseases [6]. Studies have shown that abnormal splicing is a common feature of AML and provides potential targets for AML [7–9]. Aberration in splicing contributes to the megakaryocytic malignancies. An early study demonstrated that an AS product of GATA Binding Protein 1 (GATA1) isoform which lacks the transcriptional activation domain, triggers excessive megakaryocyte proliferation [10]. More recently, it was reported that c-Mpl-del, an AS isoform of the thrombopoietin receptor c-Mpl, is upregulated in AMKL patients. This mis-spliced product promotes chemoresistance and proliferation of AMKL cells [11]. These studies indicate that the abnormalities in splicing contribute to the pathogenesis of AMKL and targeting the splicing process is a potential therapeutic strategy for AMKL treatment.

RBM39 is an RNA-binding protein (RBP), as well as a pre-mRNA splicing factor and transcription coactivator [12–14]. When working as components of splicesome, RBM39 interacts with other splicing factors, including U2AF65, SF3b155 et al., to regulate pre-mRNA alternative splicing [15–17]. Increasing evidence supports that splicing homeostasis maintained by RBM39 was essential in tumor progression, which makes RBM39 a promising target of cancer [18–21]. The aryl sulfonamide drug indisulam (also known as E7070) selectively targeting RBM39 is initially identified by Eisai during screening for small molecule inhibitors that target cell cycle progression [22, 23]. Mechanistically, indisulam promotes the interaction between RBM39 and DCAF15 E3 ligase substrate receptors, leading to the ubiquitination and proteasome-mediated degradation of RBM39 [24], which belongs to the U2AF-like RBP family and participates in tumorigenesis and developmental processes [25–27] by regulating AS, transcription, and translation [28]. Its efficacy and safety have been demonstrated in multiple phase I [29–31] and phase II clinical trials [32–34] in patients with advanced cancers. However, the efficacy of indisulam has never been tested in AMKL.

In this study, we investigated the efficacy of RBM39 inhibitor indisulam and analyzed the role of RBM39 in cell survival in AMKL. Using RNA-seq and proteomics analysis, we demonstrated the role of RBM39 in maintaining the precise splicing in AMKL cells. Together, our study provided new clues of using RBM39 inhibitor for AMKL treatment.

## Materials and methods

### Cell lines and culture

The human tumor cell lines and 293FT cells were obtained from the Cell Bank of the Chinese Academy of Sciences. CMK, MEG01, M07e, U937, and K562 cells were cultured in RPMI medium (Vivacell, C3001-0500, Shanghai, China) supplemented with 1% penicillin/streptomycin (P/S, Beyotime, C0222 ) and 10% fetal bovine serum (FBS, Vivacell, C04001-050). In addtion, M07e cells were treated with 10 ng/ml recombinant human GM-CSF (*E.coli*) (Novoprotein, C003, Suzhou, China). 293FT cells were cultured in high-glucose DMEM medium (Vivacell, C3113-0500) supplemented with 1% P/S and 10% FBS. All the cell lines were maintained in a humidified atmosphere (37℃, 5%CO_2_). All the cell lines were subjected to short tandem repeat (STR) identification, and the identification results are shown in Supplementary Material 1.

### Cell counting kit-8 (CCK-8) assay

To assess the effect of indisulam on cell viability, CMK, MEG01, M07e, U937, and K562 cells were seeded in 96-well plates at a density of 1 × 10^4^ cells/well. Indisulam (MedChemExpress, HY-13650, NJ, USA) was dissolved and diluted with dimethyl sulfoxide (DMSO, Sigma-Aldrich, D2650, MO, USA). After indisulam treatment for 72 h, cell proliferation ability and viability were detected via a CCK-8 kit (APExBIO, K1018, TX, USA) according to the manufacturer's instructions. Each concentration was tested in three independent experiments. The short hairpin RNA (shRNA)-treated cells were seeded in 96-well plates at a density of 1 × 10^3^ cells/well and were tested every two days for a total of one week after puromycin selection. The absorbance at 450 nm was measured with a spectrometer (Thermo, MA, USA), and the ability of proliferation and viability were quantified via Graph Prism 9.0 (GraphPad Software Inc., San Diego, CA, USA). Three technical replicates were performed for each experiment.

### Soft agar colony formation

To detect the proliferative ability and invasiveness, the cells were treated or transduced as described above and 1 × 10^4^ cells/well were inoculated in a soft agar medium seeded into 6-well plates. Every 1 to 2 days, 100 to 200 μl complete medium was added to support nutrients and prevent gel cracking. After approximately 2 to 3 weeks, the cells were fixed overnight with 4% paraformaldehyde (Beyotime, P0099). The samples were subsequently stained with Giemsa solution (Beyotime, C0131) and photographed. Finally, the number of colonies in each group was calculated as the number of cells that formed colonies $\div$ the total number of cells × 100%. The data are representative of 3 technical replicates.

### Lentivirus preparation and infection

The shRNAs targeting RBM39 and ZMYND8 were inserted into the pLKO.1-puro lentiviral vector (IGE Biotechnology Ltd., Guangzhou, China) containing puromycin resistance. To prepare lentiviruses, we purchased the envelope plasmid and packaging plasmid from Addgene (pMD2. G: #12259; psPAX2: #12260; Cambridge, MA, USA). Then, we transfected the purified plasmids together with pMD2. G and psPAX2 into 293FT cells via polyethyleneimine (PEI) (Sigma-Aldrich, 49553-93-7) at a ratio of 4:1:3 according to the manufacturer’s protocol. After 6 h of transfection, replace the medium with fresh medium. The supernatants were collected at 48 h and 72 h after transfection and filtered through a 0.45 μm filter. To concentrate the lentivirus, a quarter volume of PEG8000 (Sigma-Aldrich, P5413) was then mixed with lentivirus and incubated on a shaker at 4 °C. After 24 h, the samples were centrifuged at 4000 *g* for 20 min. The resulting pellet was finally resuspended by small volume of Phosphate-buffered saline (PBS). When performing lentivirus transfection, AMKL cells were incubated with concentrated lentivirus in the presence of 1 μg/mL hexadimethrine bromide (Polybrene) (Sigma-Aldrich, H9268) for 24 h. After transfection, stable cell lines were selected by puromycin (Beyotime, ST551) for 3 days. The sequences of shRNA are listed in Supplementary Table S1.

### Real-time quantitative PCR (RT-qPCR)

To detect the mRNA expression level, RNA was extracted via a Total RNA Isolation Kit (Vazyme, RC112-01; Nanjing, China) according to the manufacturer's instructions. Total RNA was then reverse transcribed into cDNA via a reverse transcription kit (Applied Biosystems, 94404, CA, USA). RT-qPCR was performed via LightCycler 480 SYBR Green I Master Mix (Roche, 4887352001, Basel, Switzerland) in a LightCycler 480 Real-Time System (Roche). The relative mRNA expression of the target genes was subsequently calculated via the ${2}^{\Delta \Delta \text{Ct}}$ method, with glyceraldehyde-3-phosphate dehydrogenase (GAPDH) expression used as a control. Three technical replicates were performed for each experiment. The primers used in this study are shown in Supplementary Table S2.

### RNA splicing analysis and verification

Following the alignment of the RNA-seq data, rMATS (4.1.2) software was used to analyze alternative splicing events, including skipped exons (SEs), retained introns (RIs), mutually exclusive exons (MXEs), alternative 5' splice sites (A5SSs), and alternative 3' splice sites (A3SSs). The splicing events were visualized via the IGV Genome browser (version 2.19.2). Significant splicing events were subsequently chosen based on the criteria of a false discovery rate (FDR) < 0.05 and an absolute difference in inclusion level (IncLevel) > 0.2. Total RNA from vehicle- or indisulam- treated cells were extracted by fastPure Cell/Tissue Total RNA Isolution Kit V2 (Vazyme, RC112-01; Nanjing, China). Then, cDNA was synthesized from 1000 ng of total RNA using the reverse transcription kit (Applied Biosystems, 94404, CA, USA) in a 25 μl reaction on an ABI PCR instrument (Thermo Fisher, Applied Biosystems). To confirm the RNA splicing events, PCR was performed to detect SE of EZH2 and MXE of ZMYND8. cDNA was amplified using 2 × Taq Plus Master Mix II (Vazyme, P213) and subjected to electrophoresis on 1.2% agarose gel at 130 V for 30 min. Finally, the gel was visualized using a gel imager (UVITECT, FireReader, UK) under ultraviolet light. The PCR primers used are listed in Supplementary Table S2.

### Western blot analysis

On the first day, AMKL cells were collected after the corresponding treatment and washed with PBS before lysis using RIPA lysis buffer (Beyotime, P0013B). The extracted protein concentration was quantified to be 10 mg/ml. Proteins of the same mass were separated via electrophoresis, transferred to polyvinylidene fluoride (PVDF) membranes (GVS, 1212639, Zola Predosa, Italy), and blocked with 5% skim milk. Then, primary antibodies were added, and the samples were incubated overnight at 4 °C with shaking. On the second day, the PVDF membranes were incubated with secondary antibodies at room temperature for 1 h. After the PVDF membranes were wetted with ECL luminescence solution (Millipore, WBKLS0500, USA), the membranes were visualized with an AI600 image gel imaging analyzer (GE, MA, USA). GAPDH was used as a loading control. The raw images of western blots are provided in the supplementary material. The antibody details are shown in Supplementary Table S3.

### RNA sequencing (RNA-seq)

To detect the mechanically induced effects of indisulam on AMKL, CMK cells treated with indisulam were collected and sent to Novogene Bioinformatics Technology Co., Ltd. (Beijing, China) for sequencing. Libraries were constructed from total RNA, and the resulting mRNA fragments were used as templates to synthesize the first strand of cDNA via the M-MuLV reverse transcriptase system, followed by the second strand of cDNA from dNTPs. The purified and screened cDNAs were subjected to PCR amplification, and the resulting products were subsequently purified to obtain a library. DESeq2 software (version 1.40.0) was used for differential expression analysis between the two comparison combinations. The sequencing results are shown in Supplementary Table S4.

### Quantitative proteomics

#### Protein extraction

Proteomic analysis was performed by Jingjie Biotechnology Co., Ltd. (Hangzhou, China). Samples were suspended in lysis buffer (8 M urea, 1% protease inhibitor) and lysed by ultrasonic disruption. The total protein was collected by centrifuge at 4 °C, 12,000 *g* for 10 min and proceed to concentration determination using BCA assay.

#### Trypsin digestion

For digestion, equal amounts of protein were taken from each sample and adjusted to the same volume with lysis buffer. Trichloroacetic Acid was added to a final concentration of 20%, vortexed, and incubated at 4 °C for 2 h to precipitate proteins. After centrifugation (4500*g*, 5 min), the supernatant was discarded, and the pellet was washed 2–3 times with cold acetone. After drying the precipitate, 200 mM Triethylammonium Bicarbonate was added to reach the final concentration. The precipitate was sonicated to disperse, and trypsin was added at a 1:50 (protease:protein, m/m) ratio for overnight digestion. Dithiothreitol was added to a final concentration of 5 mM, and reduction was carried out at 56 °C for 30 min. Iodoacetamide was then added to a final concentration of 11 mM, and the mixture was incubated at room temperature in the dark for 15 min.

### LC–MS/MS analysis

The peptides were dissolved in mobile phase A of the liquid chromatography system and loaded onto the Evotip according to the manufacturer's instructions. The peptides were then separated using the Evosep One ultra-high-performance liquid chromatography system, utilizing the pre-set 60 SPD method. After separation, the peptides were ionized in the Capillary ion source and injected into the timsTOF Pro 2 mass spectrometer for data acquisition. The data were acquired using the data-independent parallel accumulation serial fragmentation mode. FDR was adjusted to < 1%. The proteins with a *P*-value < 0.01 and a log2-fold change between the two groups, either < − 0.9 or > 1.11, were considered significantly different in expression. These significant proteins were then selected and verified via Western blot analysis.

### Apoptosis and cell cycle assays

We collected AMKL cells and washed them with precooled PBS. Then, the cells were suspended in 1 × binding buffer and stained with fluorescein isothiocyanate (FITC)-Annexin V antibody (BD Biosciences, 556420, NJ, USA) and propidium iodide (PI) solution (BD Biosciences, 556463) for apoptosis analysis. For cell cycle analysis, the cells were harvested, washed with precooled PBS, and fixed in 75% ethanol for 24 h. The cells were processed via the Cell Cycle and Apoptosis Assay Kit (Beyotime, C1052) following the manufacturer's instructions. Both the apoptosis and cell cycle data were processed via flow cytometry (Beckman Gallios™ Flow Cytometer; Beckman, USA). Each experiment was performed three technical replicates.

### Generation of CRISPR–Cas9 DCAF15 knockout

The Cas9 gene was introduced into CMK and MEG01 cell lines through lentiviral transduction and selected with 500 μg/mL geneticin (MedChemExpress, HY-108718) for one week to establish stable Cas9-expressing cell lines. Small guide oligos (sgRNAs) targeting DCAF15 were then cloned and inserted into the Lenti-CRISPR plasmid, which was purchased from GENECHEM (Shanghai, China). Cells expressing the Cas9 gene were transduced with either sgRNA-DCAF15 or nontargeting control sgRNA (sgNC) lentiviral particles. Lentivirus preparation was conducted by GENECHEM via previously established protocols. The sequences of the sgRNAs are listed in Supplementary Table S1.

### In vivo experiments

All experiments related to animals were approved by the ethics committee of the Animal Care Committee of Soochow University (CAM-SU-AP#: JP-2018-1). NSG female mice aged 4–6 weeks were randomly divided into two or three groups. One hundred microliters of a cell suspension prepared in PBS (1 × 10^6^ CMK cells expressing firefly luciferase) was injected into each mouse via the tail vein. For drug treatment, indisulam was dissolved in 20% SBE-β-CD (MedChemExpress, HY-17031) in saline and administered via intraperitoneal injection (12.5 mg/kg/day) or vehicle for 7 days. After 10 days of injection, in vivo imaging was conducted by an imaging system (Berthold, NightOWL II LB 983, Germany) every 2 to 3 days. The fluorescence intensity, body weight, adverse skin events, hair condition, and incidence of diarrhea were monitored during the treatment period. At the end of the study, the paraffin-embedded tissue blocks from the liver, spleen, and hind limbs were prepared for hematoxylin and eosin (HE) staining and immunohistochemistry (IHC) with a Ki67-antihuman antibody (Servicebio, GB12114, China). All mice were euthanized before reaching a 20% body weight loss.

### Statistical analysis

Statistical analysis was performed using GraphPad Prism software (version 9.0) and R (version 4.2.2). The Mann–Whitney U test was used to compare the two groups' differences, and the Pearson method was used for correlation analysis. In the case of three groups, one-way ANOVA was performed. The survival time was analyzed using the Log-Rank test with *P* < 0.05 considered statistically significant. The following symbols indicated the data significance: * *P*< 0.05, ** *P* < 0.01, *** *P* < 0.001, and **** *P* < 0.0001.

## Results

### Indisulam effectively inhibits survival of AMKL cells

**Fig. 1:**
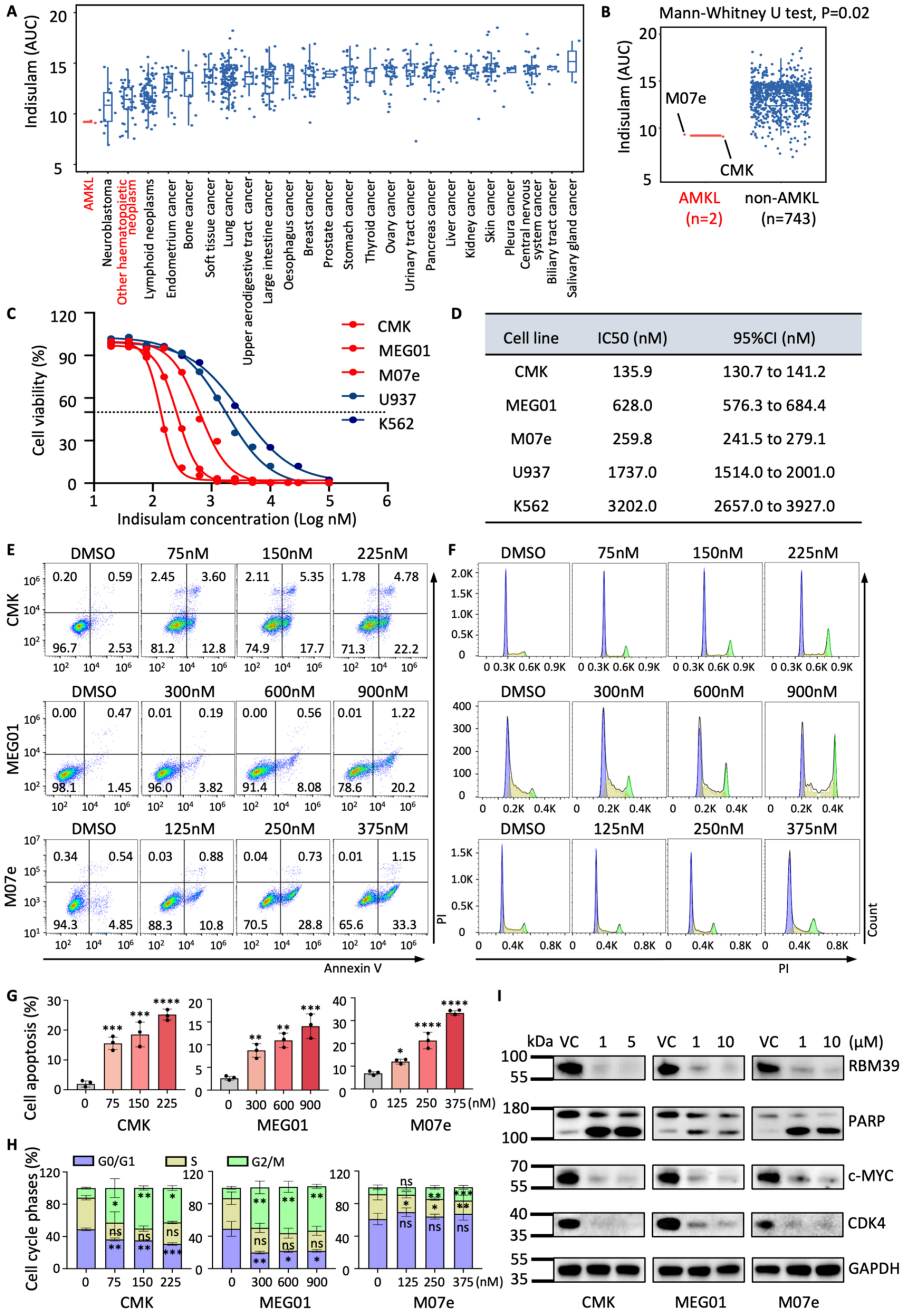
AMKL cells are highly sensitive to indisulam. **A** Median area under the curve (AUC) of mortality in 25 types of tumors. **B** AUC comparisons between AMKL cell lines (CMK and M07e cell lines, n = 2) and non-AMKL cell lines (all other tumor cell lines, n = 743). Data were acquired from the Cancer Target Discovery and Development (CTD^2^) Network, each dot represents a cell line. **C** Dose–response curves of AMKL (CMK, MEG01, M07e) and other AML subtypes (U937 and K562) treated with indisulam. Cell viability was measured by a CCK-8 assay. **D** IC50 values and 95% confidence intervals (95% CIs) of indisulam in each AML cell line. **E** Flow cytometry analysis of Annexin V + cells after indisulam treatment at different concentrations. **F** Flow cytometry analysis of the cell cycle after indisulam treatment at different concentrations. **G** Statistical plots of the proportion of cells with indisulam-induced apoptosis. **H** Compared with DMSO treatment, indisulam treatment resulted in strong G2 phase arrest in AMKL cells. **I** Western blot detection of PARP, c-MYC, and CDK4 expression after indisulam treatment in AMKL cell lines. The error bars denote the Standard Deviation (SD). *P* values were determined via Mann–Whitney U test and are indicated as *P < 0.05, **P < 0.01, ***P < 0.001, and ****P < 0.0001. "ns" signifies not significant. VC = vehicle control, 0.1% DMSO. Each experiment was performed with three technical replicates

Nijhuis et al. have shown that leukemia cells exhibited excellent sensitivity to indisulam [35]. We focused on the AMKL subtype of leukemia and found AMKL showed the greatest sensitivity among 745 tumor cell lines (Fig. 1A, B). The half-inhibitory concentration (IC50) values of indisulam in AMKL cells (CMK, MEG01, and M07e) were significantly lower than those in non-AMKL cells (U937 and K562) (Fig. 1C, D). Moreover, indisulam treatment inhibited the growth of AMKL cells and reduced cell survival (Fig. S1). The proportion of apoptotic cells increased in a dose-dependent manner (Fig. 1E, G). Cell cycle analysis revealed that AMKL cells exhibited a decrease in the number of cells in the G1 phase and an increase in the number of cells in the G2 phase after indisulam treatment (Fig. 1F, H). Finally, we confirmed that indisulam treatment led to degradation of the RBM39 protein, cleavage of the classical apoptotic protein poly ADP-ribose polymerase (PARP), and decreased expression levels of c-MYC and the classical cell cycle protein Cyclin-dependent kinase 4 (CDK4) (Fig. 1I). These findings confirmed that AMKL is highly sensitive to indisulam. Indisulam-induced RBM39 degradation leads to cell cycle arrest and apoptosis in AMKL cells.

### Knockdown of RBM39 recapitulates the effects of indisulam

**Fig. 2:**
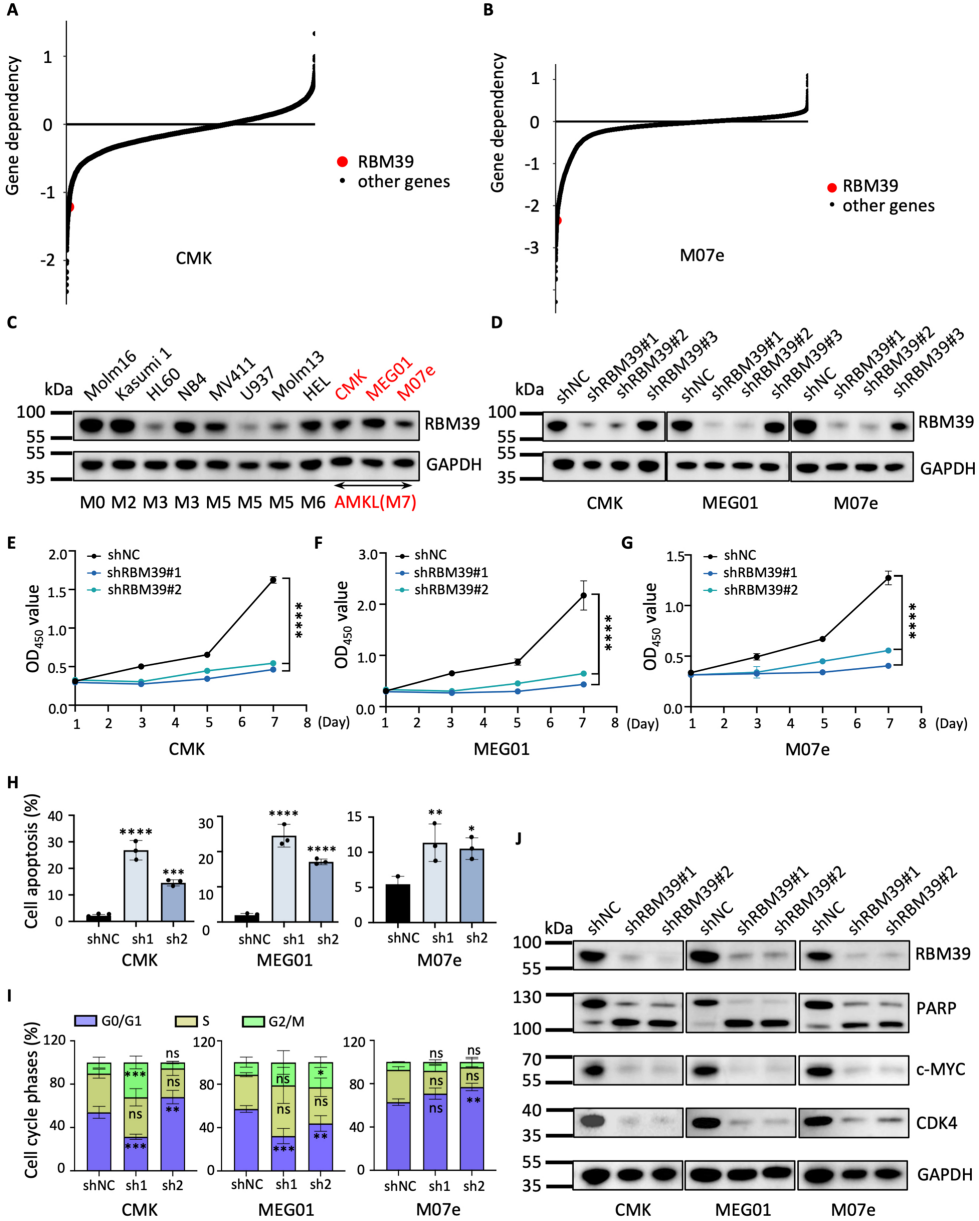
RBM39 is highly expressed in AMKL and promotes AMKL cell survival. **A**, **B** CRISPR screen data indicates the gene dependency of RBM39 in CMK and M07e cells. **C** Western blot analysis showing the protein expression of RBM39 in AML cells. **D** Western blot analysis showing the knockdown efficiency of RBM39 in AMKL cells. **E**–**G** CCK8 assay showing the proliferation curves of CMK, MEG01, and M07e cells transduced with shRBM39#1, shRBM39#2 or shNC. **H** Quantification of apoptotic AMKL cells after RBM39 knockdown using AnnexinV/PI dual staining. **I** The bar graphs show the proportion of AMKL cells at each cell cycle phase after RBM39 knockdown. **J** Western blot analysis of the PARP, c-MYC, and CDK4 proteins after RBM39 knockdown in AMKL cell lines. The error bars denote the SD. *P* values were determined via Mann–Whitney U test and are indicated as *P < 0.05, **P < 0.01, ***P < 0.001, and ****P < 0.0001. "ns" signifies not significant. Each experiment was performed with three technical replicates

As the target of indisulam, the role of RBM39 in AMKL has not yet been clarified. DEMETER2 is a framework that evaluates gene dependencies across 712 cancer cell lines and was examined in three distinct comprehensive pooled RNA interference screens. By using this platform, we revealed a high dependency of RBM39 in 2 AMKL cell lines (CMK and M07e), underscoring an indispensable role of RBM39 in AMKL cell survival (Fig. 2A, B). We then investigated RBM39 expression patterns in AML patients via the Sangerbox web tool (a bioinformatics data analysis platform) via The Cancer Genome Atlas (TCGA) database, which revealed increased RBM39 expression in AML samples compared with normal samples (Fig. S2A, B), and high RBM39 expression was correlated with an unfavorable prognosis in AML patients (Fig. S2C). Additionally, our experimental results confirmed that RBM39 was highly expressed in AMKL cell lines (Fig. 2C, S2D). The shRNA-mediated knockdown of RBM39 expression in AMKL (Fig. S2E) significantly reduced the cell proliferation rate (Fig. 2E–G), promoted apoptosis (Fig. 2H, S2F), and disrupted cell cycle progression (Fig. 2I, S2G). Finally, knockdown of RBM39 induced cleavage of PARP, decreased c-MYC, and CDK4 expression, confirming the role of RBM39 in regulating proliferation, apoptosis, and the cell cycle in AMKL cells (Fig. 2J). Taken together, our results indicate that RBM39 is essential for the growth and survival of AMKL cells.

### RBM39 knockdown delays AMKL development in vivo

**Fig. 3:**
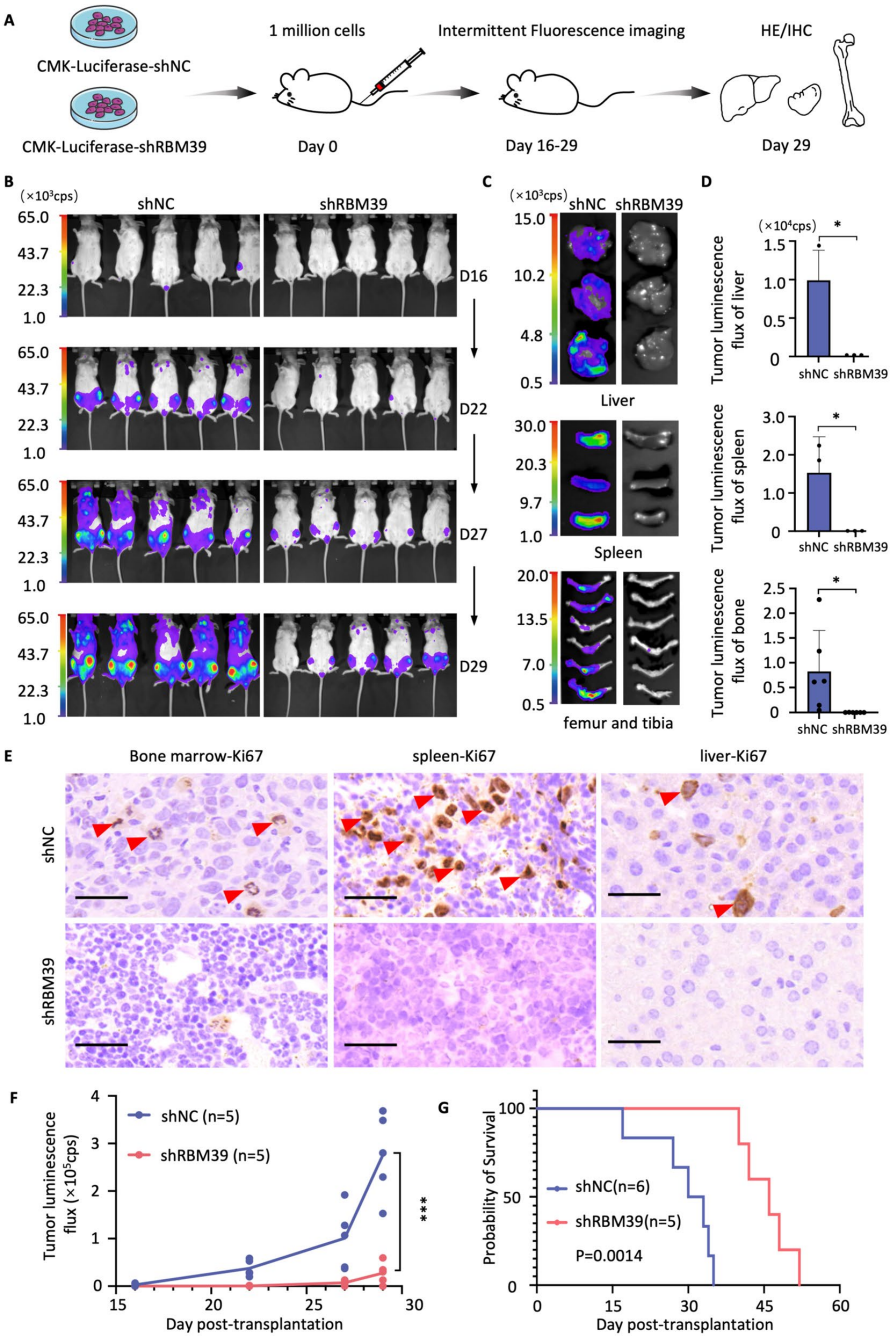
Knockdown of RBM39 expression delayed the leukemia development in the AMKL mouse model. **A** Scheme of the mouse xenograft experiment using CMK-luciferase cells transduced with shNC (n = 11) or shRBM39 lentivirus (n = 10). "n" refers to biological replicates. **B** Representative bioluminescence images of NSG mice transplanted with CMK-luciferase-shNC or CMK-luciferase-shRBM39 cells. **C** The tumor fluorescence signal intensity in the liver (upper), spleen (middle), femur and tibia (lower). **D** Statistical analysis of tumor fluorescence signal intensity. **E** IHC (Ki67) staining of the bone marrow, spleen, and liver tissue sections. Ki67-positive cells are indicated by red arrows. **F** Line graph showing the tumor burden based on bioluminescence imaging. "n" refers to biological replicates. **G** Kaplan–Meier analysis of CMK xenografts suggested that the shRBM39 group had a significantly prolonged survival time compared with the shNC group. "n" refers to biological replicates. The error bars denote the SD. *P* values were determined via Mann–Whitney U test and are indicated as **P < 0.01 and ***P < 0.001. "ns" signifies not significant. The survival time was analyzed by the Log-Rank test, and *P* < 0.05 was considered statistically significant

To evaluate the role of RBM39 in vivo*,* we intravenously injected CMK-luciferase-shNC cells (negative control group) or CMK-luciferase-shRBM39 cells (knockdown group) into NSG mice (Fig. 3A). The knockdown group presented a significantly reduced overall leukemia burden (Fig. 3B–D, S3A, B). There were no significant changes in body weight (Fig. S3C) or pathological changes in the appearance of the liver or spleen (Fig. S3D). Histological examination with HE staining (Fig. S3E) and IHC confirmed reduced leukemia cell infiltration in the bone marrow, liver, and spleen in the RBM39-knockdown group (Fig. 3E, F, S3F). Overall survival was significantly prolonged in the knockdown group (Fig. 3G). These findings demonstrated that RMB39 promoted AMKL development in vivo.

### RBM39 degradation leads to AS defects in AMKL cells

**Fig. 4:**
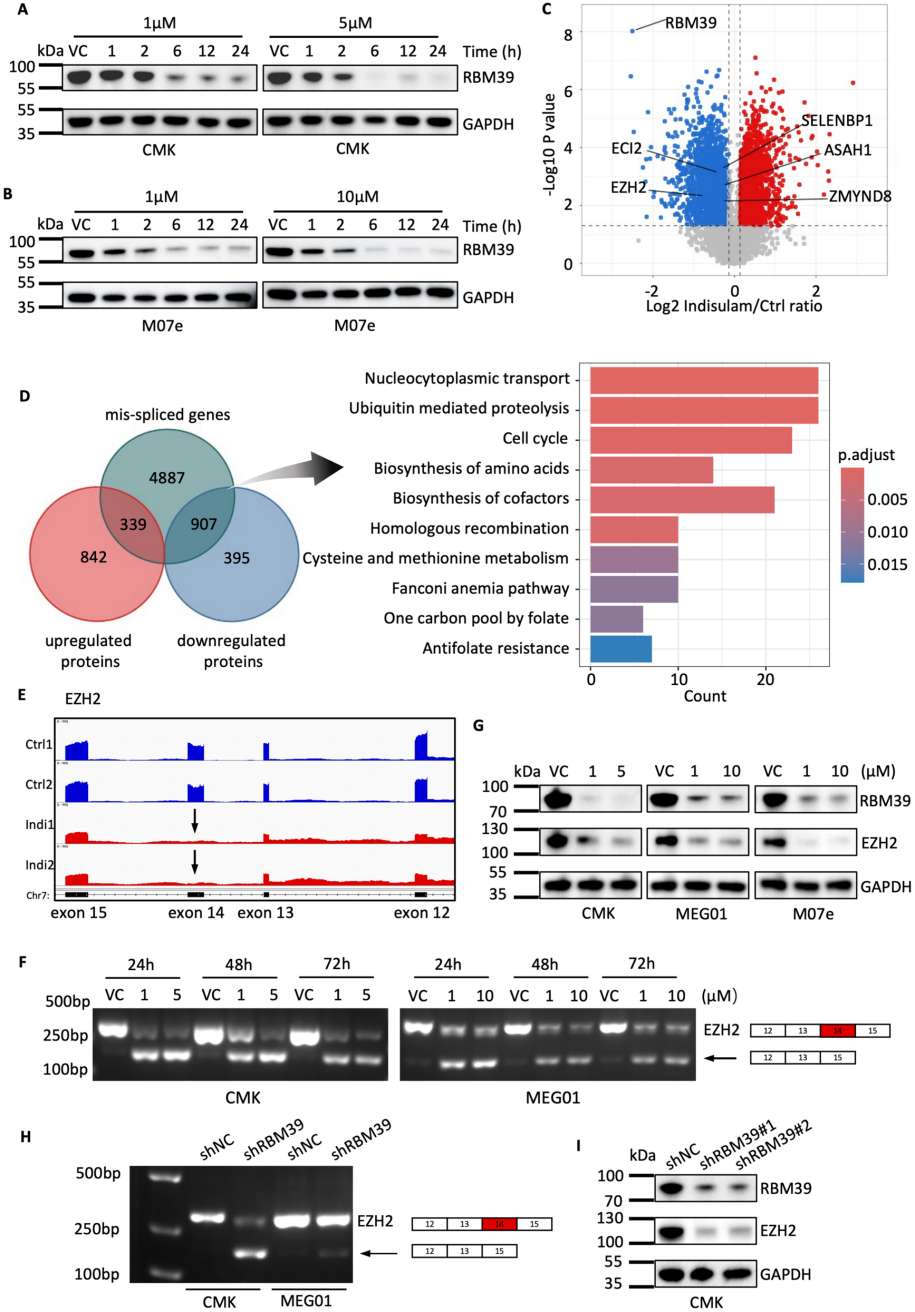
RBM39 degradation by indisulam results in the overall mis-splicing in AMKL cells. **A**, **B** Western blot analysis of RBM39 in CMK cells and M07e cells treated with indisulam at the indicated time points. **C** Volcano plot of the proteomic analysis of CMK cells treated with indisulam (5 µM) or VC for 24 h. **D** Venn diagram of mis-spliced genes versus upregulated proteins (left) and KEGG pathway analysis of the intersection between mis-spliced genes and downregulated protein genes (right). **E** The Sashimi plot depicts the SE event of the EZH2 gene region in CMK cells following indisulam treatment. The black arrows highlight the skipping of exon 14. **F** PCR analysis of EZH2 (exons 12–15) in CMK and MEG01 cells treated with VC or indisulam for 24–72 h. **G** Western blot analysis of RBM39 and EZH2 expression after indisulam treatment in AMKL cells. **H** PCR analysis of EZH2 in CMK cells with RBM39 knockdown. **I** Western blot analysis of EZH2 in CMK cells with RBM39 knockdown

RBM39 is involved in transcription regulation and RNA splicing [25], and we then investigated the mechanism by which RBM39 regulates the growth of AMKL cells. In AMKL cells, the RBM39 protein was almost completely degraded within 24 h when 5 μM indisulam was administered (Fig. 4A, B). Therefore, 5 μM indisulam-treated CMK cells were collected and subjected to proteomic assays (Fig. 4C). Transcriptome analysis revealed increased RNA splicing events, including primarily SE events and RI events, in the indisulam-treated group than in the control group (Fig. S4A, B). Among these genes, 907 mis-spliced genes were downregulated (Fig. 4D). Kyoto Encyclopedia of Genes and Genomes (KEGG) pathway enrichment analysis suggested that these 907 affected genes were enriched in ubiquitin-mediated proteolysis and cell cycle pathways (Fig. 4D). EZH2 has been identified as a critical target with splicing process defects [26]. In this study, EZH2 exhibited SE events at exon 14 in indisulam-treated CMK cells (RNA-seq read counts, IGVs; Fig. 4E). We validated the RNA splicing (Fig. 4F) and lack of protein (Fig. 4G) of EZH2, suggesting that the mis-splicing of EZH2 resulted in a defect in translation. Additionally, the knockdown of RBM39 by shRNA resulted in the same pattern of mis-splicing of EZH2 at the mRNA level and a reduction in the EZH2 protein level (Fig. 4H, I). Therefore, RBM39 deficiency leads to extensive splicing dysregulation in AMKL.

### ZMYND8 is a downstream target of RBM39 in AMKL cells

**Fig. 5:**
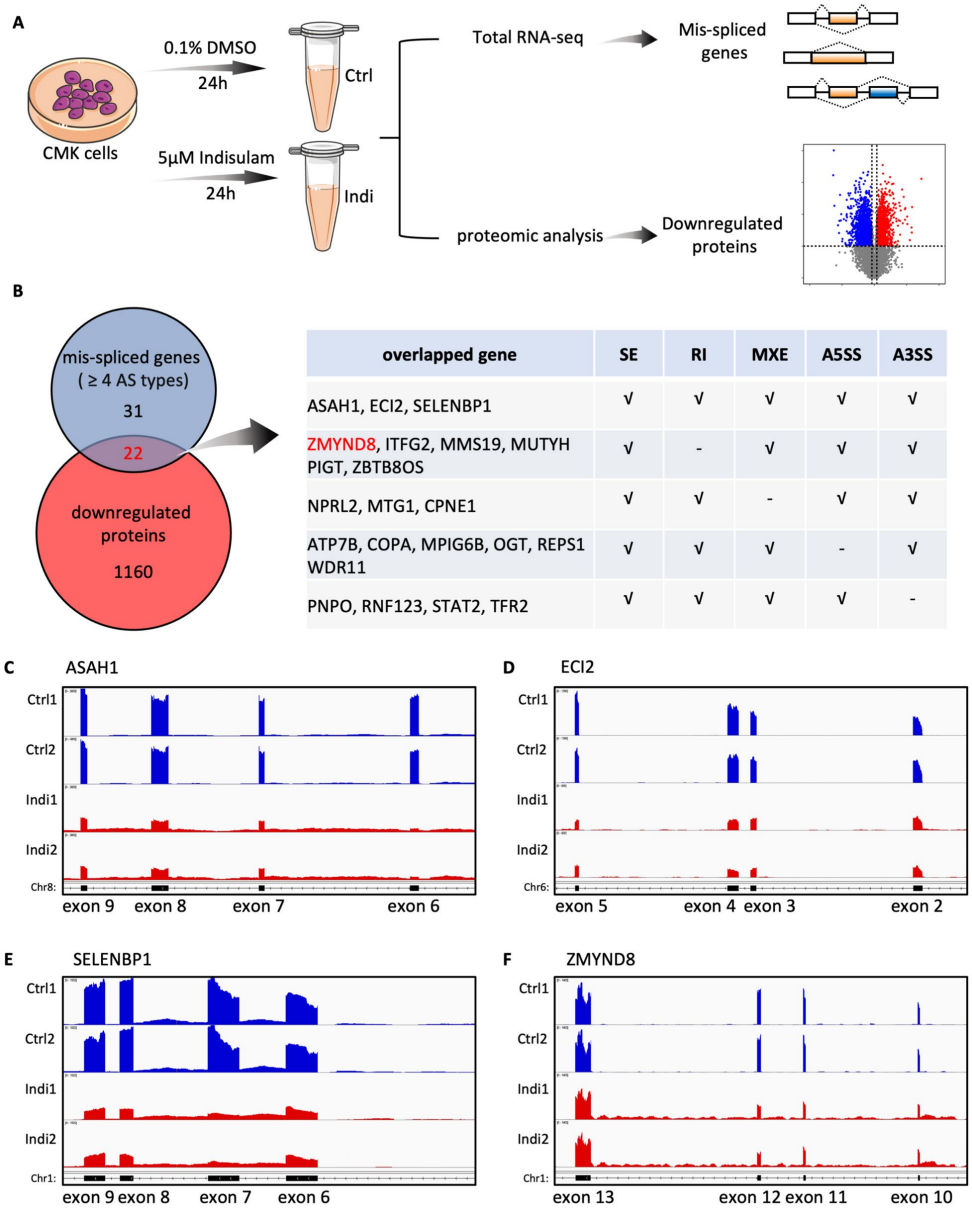
Indisulam causes aberrant splicing of important AMKL genes. **A** Schematic representation of CMK cells subjected to RNA-seq and proteomic analyses. **B** Venn diagram of mis-spliced genes (≥ 4 AS types) and downregulated proteins (*P* value < 0.01 and the ratio between the two groups < 0.9). **C–F** Sashimi diagram demonstrating the SE events of ASAH1 (**C**), ECI2 (**D**), SELENBP1 (**E**), and ZMYND8 (**F**) in indisulam-treated CMK cells

**Fig. 6:**
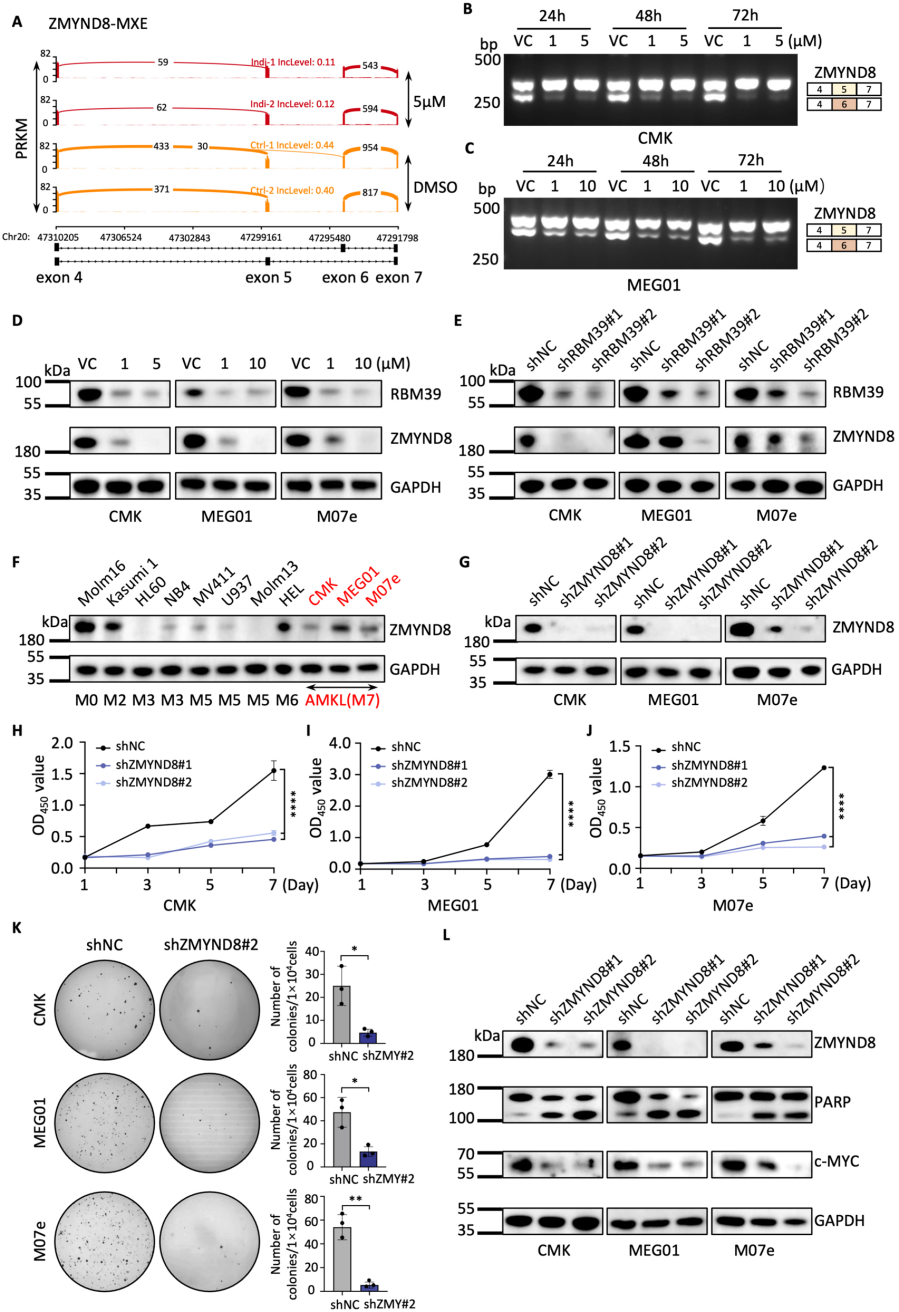
ZMYND8 is a downstream target of RBM39 in AMKL cells. **A** Sashimi plot generated via the IGV program showing the splicing changes of ZMYND8 gene in CMK cells after indisulam treatment. **B**, **C** PCR testing of ZMYND8 AS after indisulam treatment. **D** Western blot analysis of the expression of ZMYND8 after indisulam treatment. **E** Western blot analysis of ZMYND8 after RBM39 knockdown. **F** Western blot analysis of the expression levels of ZMYND8 in AML cell lines. **G** Western blot analysis of the knockdown efficiency of ZMYND8. **H**–**J** Knockdown of ZMYND8 inhibited the proliferative capacity of AMKL cells. **K** Colony formation ability of AMKL cells transduced with lentiviral shNC or shZMYND8. **L** The protein expression of cleaved PARP and c-MYC following ZMYND8 knockdown. The error bars denote the SD. *P* values were determined via Mann–Whitney U test and are indicated as **P < 0.01, ***P < 0.001. "ns" signifies not significant. Each experiment was performed with three technical replicates

To identify genes targeted by RBM39 in AMKL, we performed combined RNA-seq and proteomics analyses of indisulam-treated cells (Figs. 5A, S5A). We identified 22 key genes that underwent multiple types of AS events (Fig. 5B). Among these genes, N-Acylsphingosine Amidohydrolase 1 (ASAH1), Enoyl-CoA Delta Isomerase 2 (ECI2, or PECI), Selenium Binding Protein 1 (SELENBP1), and ZMYND8 presented complex splicing patterns, generating four or five differential splicing events (Fig. 5C–F, S5B–E). Furthermore, we compared the mRNA expression levels of these genes across different AML FAB subtypes in the TCGA database and revealed that the expression levels of these genes are relatively higher in M6 and M7 (Fig. S5F–I) than in other subtypes, suggesting that RBM39 maintains the precise splicing of genes crucial for AMKL. One previous study revealed that ZMYND8 is essential for AML proliferation [36]. Therefore, we investigated the function of ZMYND8 in AMKL. First, we found that ZMYND8 exhibited significant splicing dysregulation after indisulam treatment or RBM39 knockdown (Fig. 6A–C, S6D), leading to a reduction in the protein level (Fig. 6D, E). Second, by examining the TCGA database via the Sangerbox web tool, we observed that ZMYND8 expression was higher in AML patients than in normal controls and that high ZMYND8 expression was associated with poor prognosis (Fig. S6A, B). Notably, a positive correlation was found between the mRNA expression of ZMYND8 and RBM39 in AML patients (Fig. S6C). We subsequently explored the expression of ZMYND8 in various AML cell lines and found that it is highly expressed in AMKL cells (Fig. 6F, S6E). Furthermore, the proliferation rate of AMKL cells significantly decreased after ZMYND8 knockdown (Fig. 6H–K). Finally, western blot analysis revealed increased cleaved-PARP and decreased c-MYC after the depletion of ZMYND8 (Fig. 6L). Together, these findings indicate that ZMYND8 is a direct splicing target of RBM39 and is required for AMKL survival.

### The anti-AMKL effects of indisulam are DCAF15-dependent

**Fig. 7:**
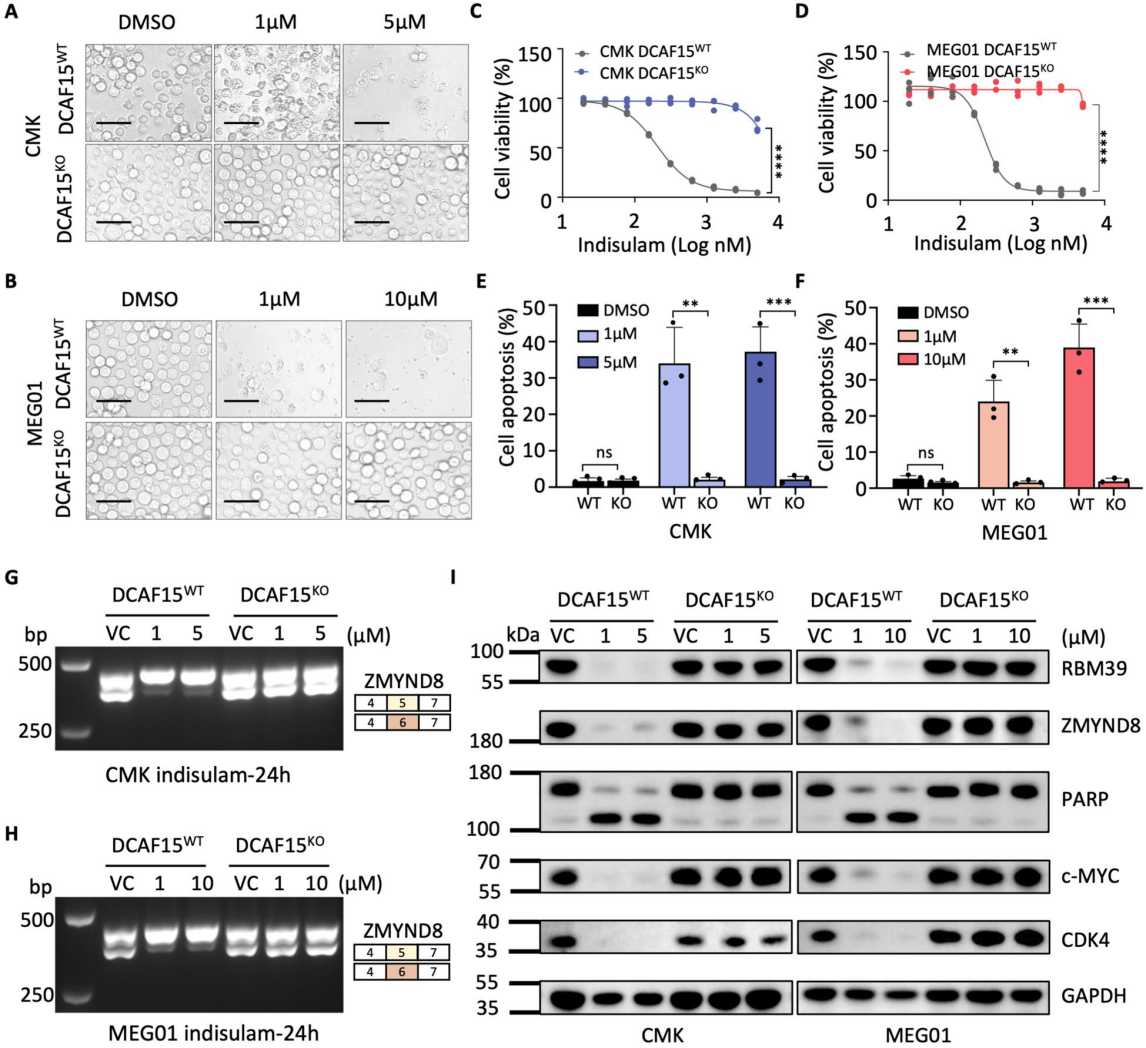
Indisulam-induced RBM39 degradation and RNA mis-splicing are DCAF15-dependent. **A**, **B** Representative images of DCAF15^WT^ or DCAF15^KO^ cells after indisulam treatment. The scale bar is 50 µm. **C**, **D** Dose–response curve of cell viability following treatment with indisulam for 72 h. **E**, **F** Comparison of apoptosis between DCAF15^WT^ and DCAF15^KO^ cells. **G**, **H** PCR analysis of ZMYND8 mis-splicing in DCAF15^WT^ and DCAF15^KO^ cells. **I** Western blot analysis of the protein expression of RBM39, ZMYND8, cleaved-PARP, c-MYC, and CDK4. The error bars denote the SD. *P* values were determined via Mann–Whitney U test and are indicated as **P < 0.01, ***P < 0.001. "ns" signifies not significant. Each experiment was performed with three technical replicates

To confirm whether DCAF15 is a key factor in the sensitivity of AMKL to indisulam, we explored DCAF15 mRNA expression levels in AML patients from the TCGA database. The results showed that the DCAF15 expression levels in AMKL (M7) were greater than those in other AML subtypes (Fig. S7A). Furthermore, we employed the CRISPR/Cas9 system to knock out DCAF15 in the CMK and MEG01 cell lines. The knockout of DCAF15 greatly reduced the cytotoxicity of indisulam to AMKL cells (Fig. 7A, B), as there was no effect on cell viability (Fig. 7C, D) or apoptosis (Fig. 7E, F, S7B, C). In addition, indisulam treatment did not induce the AS of ZMYND8 (Fig. 7G, H) or EZH2 (Fig. S7D) in the absence of DCAF15. Similarly, western blot analysis verified these findings; in the absence of DCAF15, indisulam could not induce the degradation of RBM39. Consequently, the ZMYND8, PARP, c-MYC, CDK4, and EZH2 proteins did not change (Fig. 7I, S7E). Collectively, these data indicate that indisulam exerts its anti-AMKL effect through DCAF15.

### Indisulam impairs tumor growth in vivo

**Fig. 8:**
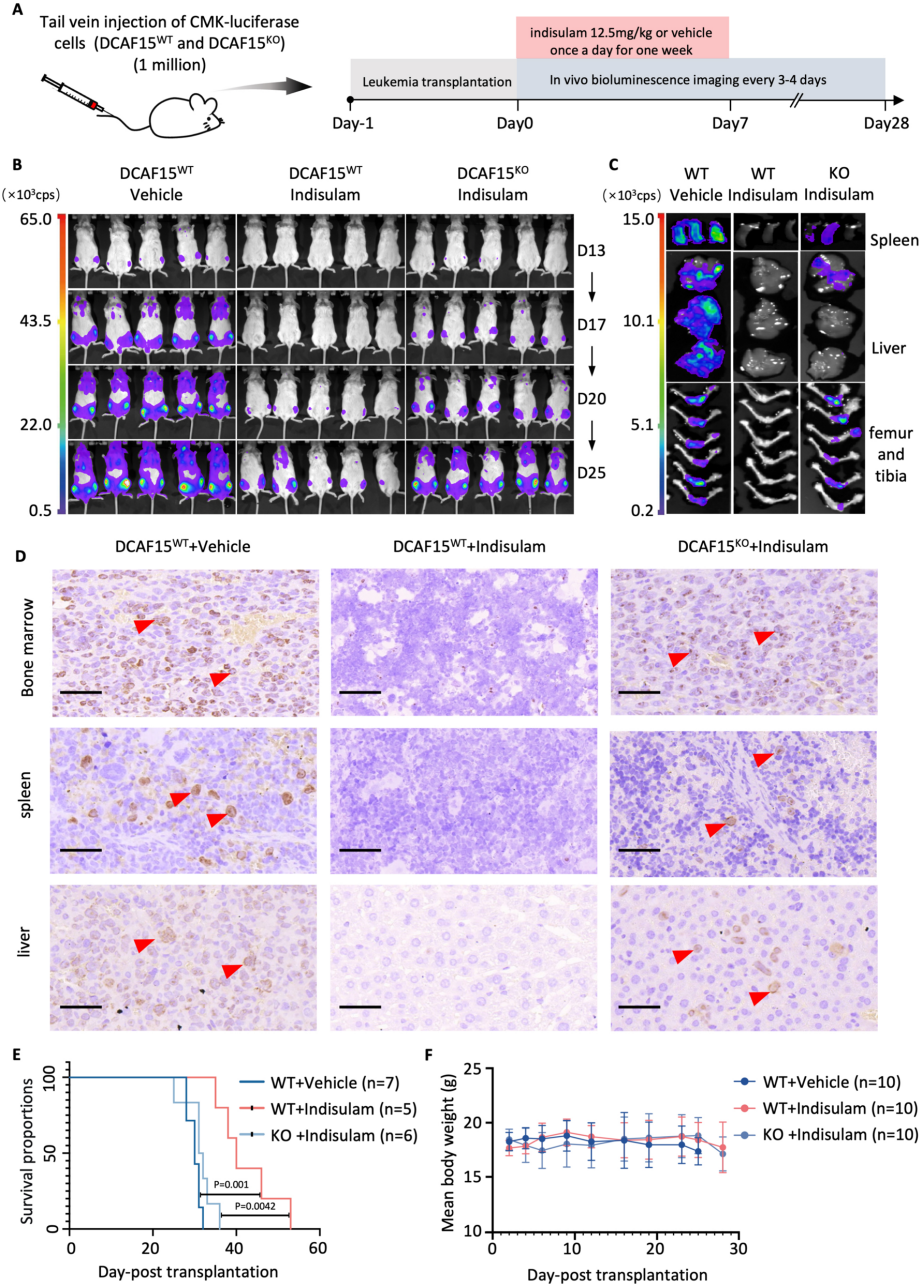
Efficacy of indisulam in a xenograft mouse model. **A** Schematics of drug treatment in AMKL mice. **B** Representative bioluminescence images of NSG mice treated with indisulam at the indicated posttransplant times. **C** The tumor fluorescence signal intensity in the spleen (upper), liver (middle), femur and tibia (lower). **D** Representative Ki67-IHC staining images from bone marrow (upper), spleen (middle), and liver (lower) tissue sections (the scale bar represents 50 μm). Ki67-positive cells are indicated by red arrows. **E** Kaplan–Meier survival curves of DCAF15^WT^ + vehicle, DCAF15^KO^ + indisulam, and DCAF15^WT^ + indisulam AMKL mice. **F** Mean body weights of the DCAF15^WT^ + vehicle group, DCAF15^KO^ + indisulam group, and DCAF15^WT^ + indisulam group. The Log-Rank test was used for survival analysis. *P* < 0.05 is considered statistically significant. "n" refers to biological replicates

To evaluate the anti-AMKL efficacy of indisulam in NSG mice, we constructed a mouse model of AMKL via luciferase-tagged CMK cells (DCAF15^WT^ or DCAF15^KO^) and randomly divided them into three groups (DCAF15^WT^ + vehicle, DCAF15^WT^ + indisulam, and DCAF15^KO^ + indisulam). AMKL mice were then administered indisulam (12.5 mg/kg/d) or vehicle for one week via intraperitoneal injection (Fig. 8A). Bioimaging revealed that indisulam effectively reduced the tumor burden in the DCAF15^WT^ group, whereas its efficacy was not observed in the DCAF15^KO^ group (Fig. 8B, C, S8A). By examining the expression of Ki67 (a proliferation marker) (Fig. 8D) and the proportion of human CD45 + (leukemia cell surface antigen) cells in the bone marrow, liver, and spleen (Fig. S8B-E), we found that the number of Ki67- and human CD45- positive cells in the DCAF15^WT^ + indisulam group was significantly lower than that in the other groups. Additionally, we found that AMKL mice in the DCAF15^WT^ + indisulam group also had better overall survival than did those in the other groups (Fig. 8E). Furthermore, we found no significant difference in body weight (for early identification of toxic reactions) (Fig. 8F) or in the appearance of the liver or spleen (Fig. S8F) among the three groups. During the treatment, no other adverse effects, such as diarrhea, hair deterioration, skin lesions, loss of appetite, or changes in movement were observed. Taken together, the in vivo results demonstrated that indisulam could effectively treat AMKL in a DCAF15-dependent manner without significant drug toxicity.

## Discussion

Spliceosome-mediated pre-mRNA editing is a critical biological process that generates mature RNAs as templates for protein synthesis. Mutations in splicing factors such as SF3B1, SRSF2, U2AF1 et al. are frequently found in AML patients [37]. Additionally, splicing factors are found to be upregulated in AML samples, confer chemo-resistance, and are associated with poor prognosis, highlighting the role of splicing factors in the pathogenesis of AML [38, 39]. Eric Wang et.al. identified a network of RBPs that are crucial for maintaining RNA splicing and the survival of AML cells. In particular, the loss of RBM39 affects RNA splicing and selectively induces lethality in AML cells harboring spliceosome mutations [20]. In our study, we demonstrated that RBM39 inhibitor indisulam exerted a profound anti-leukemic effect in AMKL-the M7 subtype of AML by inducing substantial aberrant splicing events. These findings not only underscore the importance of splicing in AMKL cell survival, but also support the oncogenic role of RBM39 in AMKL, which is consistent with research across various tumor types including AML [25], liver cancer [26], and high-risk neuroblastoma [40].

As an essential splicing factor, RBM39 regulates ~ 20% of the alternative splicing events in multiple biological processes, including cancers [41]. Disturbing the splicing of essential oncogenes by targeting RBM39 can be an effective strategy in cancers. The cancer stem cell enriched KRAS4A isoform was regulated by RBM39, thus providing treatment targeting cancer stem cells [42]. RBM39 inhibitors induced the intron retention of mRNA of homologous recombination repair (HRR) genes in cancers with homologous recombination deficiency (HRD), causing a synthetic lethality of HRD-positive tumor cells [43]. A recent study has shown that RBM39 regulated cell proliferation by affecting the splicing of MRPL33 in gastric cancer cells [44]. Our study revealed that RBM39 is responsible for maintaining the splicing of important oncogenes in AMKL cells. Many cancer-related genes [45–47], such as ZMYND8, ASAH1, and SELENBP1, were found mis-spliced following the degradation of RBM39. However, in addition to splicing factors, RBM39 can also work as a transcription factor [12, 13, 48] and a metabolic sensor [26]. In our study, several genes involved in biosynthesis of amino acids, cysteine, and methionine metabolism pathways were identified as mis-spliced following RBM39 depletion, suggestive of a possible role of RBM39 in regulating AMKL metabolism programs. The mechanism of RBM39-regulated metabolism and its association with RBM39-directed splicing events needs further investigation.

We focused on the ZMYND8, which underwent multiple forms of mis-splicing after indisulam treatment or RBM39 depletion in AMKL cells. ZMYND8 is a well-known chromatin reader which recognizes acetylated or methylated histones, and involved in transcription regulation, DNA damage and cancer [26]. ZYMND8 promotes DNA repair response by interacting with the NuRD chromatin remodeling complex [49]. Mutations or dysregulation of ZMYND8 expression are associated with cancer development and progression [50]. However, the role of ZMYND8 in cancer is still controversial. Several studies have demonstrated a tumor suppressive role of ZMYND8 by suppression of superenhancer-regulated gene expression [51, 52]. On the other hand, ZYMND8 also plays an oncogenic role in intestinal tumorigenesis via driving the enhancer-promoter interaction to upregulate the cholesterol biogenesis mevalonate (MVA) pathway [53]. In aldehyde dehydrogenase-high (ALDHhi) breast cancer stem cells (BCSC), ZYMND8 forms a positive feedback loop with NRF2 that amplifies the antioxidant defense mechanism sustaining BCSC survival and stemness [47]. Previous findings also suggested that ZMYND8 is essential for AML survival by binding to the ET domain of BRD4 through its chromatin reader domain, thereby sustaining leukemia growth [36]. In AMKL cells, we revealed that ZMYND8 was overexpressed. Knockdown of ZYMND8 suppressed AMKL cell growth and induced cell apoptosis, indicating ZMYND8 is an oncogene in AMKL.

The activity of ZMYND8 was regulated by different types of modification, such as phosphorylation [54], and acetylation [55], while the splicing of ZMYND8 was not reported. The reduced protein expression of ZMYND8 was observed after multiple splicing upon indisulam treatment. The possible mechanism could be the translation of the mis-spliced transcript was prevented by intrinsic RNA quality control steps, such as nonsense-mediated decay, non-stop decay et al. [56] or the resultant product is unstable since it is reported that the PBP (PHD-BRD-PWWP) domain of ZMYND8 was essential in binding with USP7, thereby stabilizing ZMYND8 [57]. The mechanism would be interesting to explore.

There are some limitations of this study. First, ZMYND8 underwent multiple splicing pattern changes following RBM39 deletion but the function of specific spliced products of ZMYND8 in AMKL cells was not tested in this study. Considering the eventual outcomes of multiple splicing events was the significant reduction of protein expression of ZMYND8, we demonstrated the role of ZMYND8 in AMKL cells by knockdown of the protein expression. Second, we only focused on the splicing changes of ZMYND8, while the splicing events of other genes (ASAH1 and SELENBP1) which were shown to mis-spliced following RBM39 depletion and their roles in AMKL survival were not fully demonstrated in this study. Another limitation of our study is the lack of AMKL clinical samples due to the rarity of AMKL patients. Also, the limited types of AMKL cell lines may not fully capture the complexity of AMKL in real-world clinical settings. Further validation using clinical samples and a wider range of cell lines would enhance the generalizability and robustness of our study.

## Conclusion

**Fig. 9:**
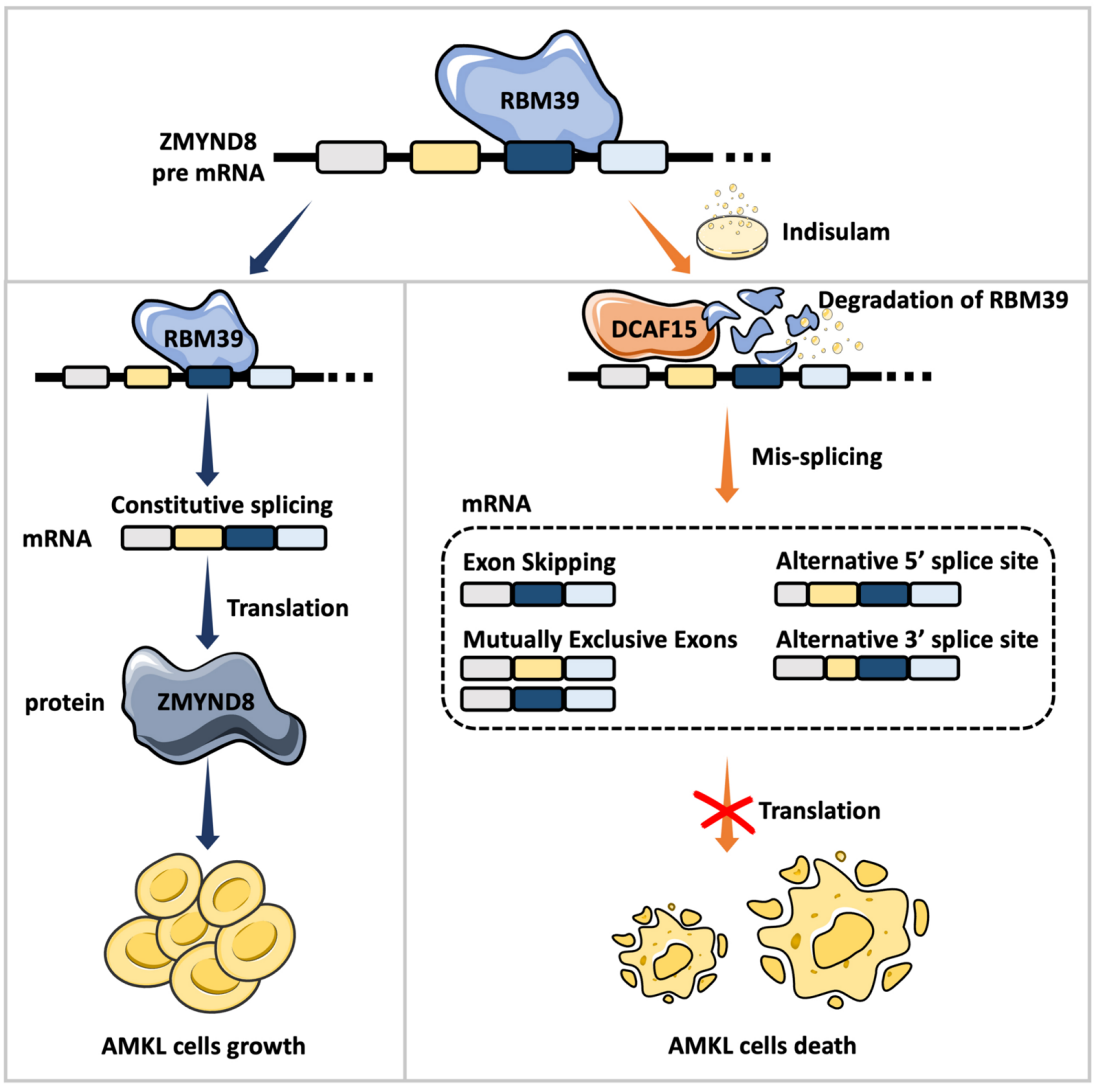
Model of potential targets of RBM39 in AMKL. Indisulam-induced degradation of RBM39 leads to alternative splicing disruption of ZMYND8 in AMKL cells, which inhibits AMKL cell growth

Together, this study demonstrated that indisulam, an RBM39 degrader, shows significant anti-AMKL activity both in vitro and in vivo. Degradation of RBM39 by indisulam induced aberrant splicing of ZMYND8 resulting in cell cycle arrest and apoptosis in AMKL cells (Fig. 9). Considering that indisulam was well tolerated by patients in Phase II clinical trials of other tumor types, our study provides potential treatment options for AMKL patients, and it could warrant further investigation in clinical trials. Additionally, the expression level of DCAF15 may serve as a biomarker for predicting sensitivity to indisulam treatment which precisely allows inclusion of AMKL patients.

## Supplementary Information

Supplementary Material 1: Fig. S1. Indisulam induces the death of AMKL cells via RBM39 protein degradation. Indisulam acts as a molecular glue that links the DCAF15 E3 ubiquitin ligase and RBM39 together, leading to polyubiquitination and degradation of RBM39 protein, and splicing defects. Representative images of AMKL cells treated for 72 h with indisulam or VC. The scale bar indicates 50 µm.

Supplementary Material 2: Fig. S2. High epression of RBM39 is associated with poor prognosis and reduced RBM39 expression by shRNA inhibited AMKL cell survival. RBM39 expression in normal tissue and tumor samples was explored via the TCGA and GTEx databases. The TCGA and GTEx databases revealed that RBM39 was upregulated in AML samples compared with normal samples. TPM, transcripts per million. T, tumor/cancer. N, normal. Kaplan–Meier survival analysis of AML patients with high or low RBM39 expression using the TCGA and GTEx databases. The difference in prognosis was significant according to the log-rank test. RT-qPCR analysis of RBM39 mRNA expression in different AML cell lines. RT-qPCR was used to detect the knockdown efficiency of RBM39 in AMKL cell lines. Flow cytometry analysis of Annexin V + cells in the shNC and shRBM39 groups. Flow cytometry analysis of apoptotic cells in the shNC and shRBM39 groups. The error bars denote the SD. *P* values were determined via Mann–Whitney U test and are indicated as *P < 0.05, ***P < 0.001, and ****P < 0.0001. "ns" signifies not significant. "N" refers to biological replicates. Each experiment was performed with three technical replicates.

Supplementary Material 3: Fig. S3. Knockdown of RBM39 led to a decreased leukemic burden in the AMKL mouse model. The leukemia burdenin the liver, spleen, and bone marrow was detected by flow cytometry. Percentages of human CD45 + cells in the liver, spleen, and bone marrow. The mean body weights of shNC- and shRBM39-treated AMKL mice. The appearance of the liver and spleen in shNC and shRBM39 AMKL mice. HE staining of bone marrow, spleen, and liver tissue sections. The error bars denote the SD. *P* values were determined via Mann–Whitney U test and are indicated as *P < 0.05, ***P < 0.001, and ****P < 0.0001. "ns" signifies not significant. Each experiment was performed with three technical replicates.

Supplementary Material 4: Fig. S4. RBM39 deletion results in altered RNA splicing. The number of AS events in CMK cells treated with 5 µM indisulam or VC for 24 h. Violin plot of the difference in the inclusion level of AS in CMK cells treated with 5 µM indisulam compared with those treated with VC.

Supplementary Material 5: Fig. S5. Indisulam treatment resulted in aberrant RNA splicing and protein changes. Violin plot of differences in protein level changes for genes with different numbers of AS types. Sashimi plots showing the aberrant RNA splicing of ASAH1, ECI2, SELENBP1, and ZMYND8.mRNA expression levels of ASAH1, ECI2, SELENBP1, and ZMYND8 in AML FAB subtypes from the TCGA database.

Supplementary Material 6: Fig. S6. ZMYND8 is highly expressed in AMKL and is associated with poor outcomes. ZMYND8 expression is higher in AML samples than in normal samples. Kaplan–Meier survival analysis of AML patients with high or low ZMYND8 expression. The difference was significant according to the Log-Rank test. Pearson correlation analysis between RBM39 and ZMYND8 mRNA expression in AML patients in the TCGA database. TPM, transcripts per million. PCR analysis of the MXE of ZMYND8 in shNC and shRBM39 CMK cells. RT-qPCR analysis of the relative expression level of ZMYND8 in different AML cell lines. RT-qPCR analysis of the knockdown efficiency of ZMYND8 in AMKL cell lines. The error bars denote the SD. *P* values were determined via Mann–Whitney U test and are indicated as *P < 0.05, **P < 0.01, and ****P < 0.0001. "ns" signifies not significant. Each experiment was performed with three technical replicates.

Supplementary Material 7: Fig. S7. DCAF15 is highly expressed in AMKL and is required for the anti-AMKL effect of indisulam. The TCGA database shows the expression of DCAF15 in AML patients based on FAB classification. Assessment of apoptosis via flow cytometry. PCR analysis of the SE of EZH2 in CMK and MEG01cells. Western blot analysis of the EZH2 protein in CMK and MEG01 cells following indisulam treatment. The flow cytometry experiments were performed with three technical replicates. Each experiment was performed with three technical replicates.

Supplementary Material 8: Fig. S8. The efficacy of indisulam was dependent on DCAF15 in a xenograft mouse model. The tumor fluorescence signal strength of the DCAF15^WT^ + Vehicle, DCAF15^WT^ + indisulam, and DCAF15^KO^ + indisulam groups. Flow cytometry was used to detect human CD45 + cells in the liver, spleen, and bone marrow of the DCAF15^WT^ + Vehicle, DCAF15^WT^ + indisulam, and DCAF15^KO^ + indisulam groups. The percentages of human CD45 + cells in the liver, spleen, and bone marrow in the three groups. The appearance of the spleen and liver in the three groups. The error bars denote the SD. *P* values were determined via Mann–Whitney U test and are indicated as *P < 0.05, ***P < 0.001, and ****P < 0.0001. "ns" signifies not significant. Each experiment was performed with three technical replicates.

Supplementary Material 9

Supplementary Material 10

## Data Availability

All data are accessible within the main text or supplementary materials. The RNA-seq data generated in this study have been submitted to the Gene Expression Omnibus (GEO) database under accession number GSE266925 with the reviewer token clapmowitfkxzod. All data generated or analysed during this study are included in this published article and its supplementary information files.

## Abbreviations

AMKL: Acute megakaryoblastic leukemia
AS: Alternative splicing
RBM39: RNA binding motif protein 39
ZMYND8: Zinc finger MYND-type containing 8
DCAF15: DDB1- and Cul4- Associated Factor 15
AML: Acute myeloid leukemia
FAB: French American British
GATA1: GATA Binding Protein 1
RBP: RNA binding protien
P/S: Penicillin/streptomycin
FBS: Fetal bovine serum
STR: Short tandem repeat
CCK-8: Cell Counting Kit-8
shRNAs: Short hairpin RNAs
PEI: Polyethyleneimine
PBS: Phosphate-buffered saline
Polybrene: Hexadimethrine bromide
RT-qPCR: Real-time quantitative PCR
GAPDH: Glyceraldehyde-3-phosphate dehydrogenase
SE: Skipped exon
RI: Retained intron
MXE: Mutually exclusive exon
A5SS: Alternative 5' splice site
A3SS: Alternative 3' splice site
FDR: False discovery rate
IncLevel: Inclusion level
PVDF: Polyvinylidene fluoride
RNA-seq: RNA sequencing
FITC: Fluorescein isothiocyanate
PI: Propidium iodide
sgRNA: Small guide oligos
HE: Hematoxylin and eosin
IHC: Immunohistochemistry
IC50: Half-inhibitory concentration
PARP: Poly ADP-ribose polymerase
CDK4: Cyclin-dependent kinase 4
TCGA: The Cancer Genome Atlas
KEGG: Kyoto Encyclopedia of Genes and Genomes
EZH2: Enhancer of zeste homolog 2
ASAH1: N-Acylsphingosine Amidohydrolase 1
ECI2: Enoyl-CoA delta isomerase 2
SELENBP1: Selenium binding protein 1
AUC: Area under the curve
SD: Standard deviation
VC: Vehicle control
