# Supplementary figures and images for: The RBM39 degrader indisulam inhibits acute megakaryoblastic leukemia by altering the alternative splicing of ZMYND8
Source: Cell Biosci. 2025 Apr 13;15:46. doi: 10.1186/s13578-025-01380-3 (PMC11995665; doi:10.1186/s13578-025-01380-3)

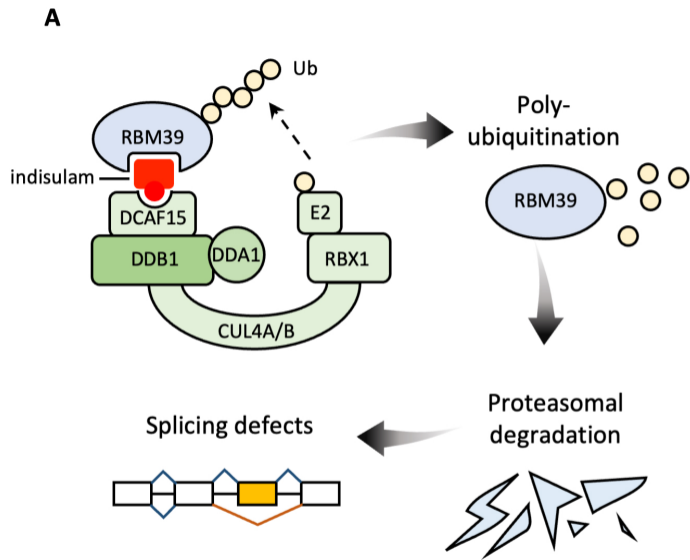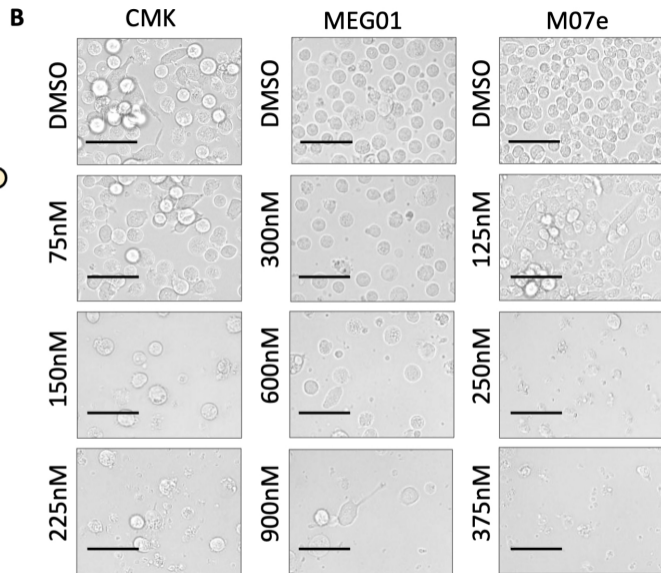

Supplement: Supplementary file 1 — Supplementary Material 1: Fig. S1. Indisulam induces the death of AMKL cells via RBM39 protein degradation. Indisulam acts as a molecular glue that links the DCAF15 E3 ubiquitin ligase and RBM39 together, leading to polyubiquitination and degradation of RBM39 protein, and splicing defects. Representative images of AMKL cells treated for 72 h with indisulam or VC. The scale bar indicates 50 µm. [file 13578_2025_1380_MOESM1_ESM.pdf]

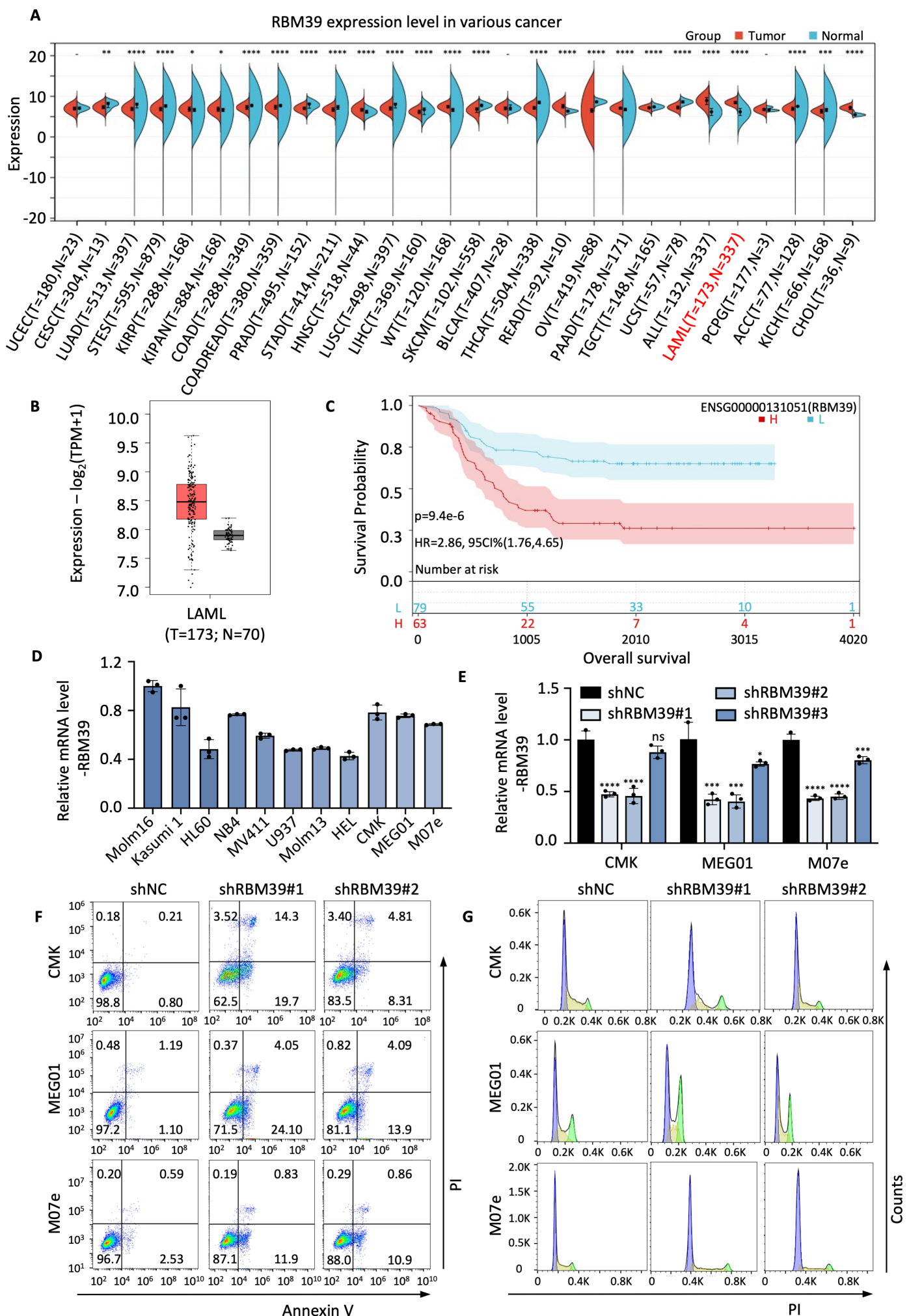

Supplement: Supplementary file 2 — Supplementary Material 2: Fig. S2. High epression of RBM39 is associated with poor prognosis and reduced RBM39 expression by shRNA inhibited AMKL cell survival. RBM39 expression in normal tissue and tumor samples was explored via the TCGA and GTEx databases. The TCGA and GTEx databases revealed that RBM39 was upregulated in AML samples compared with normal samples. TPM, transcripts per million. T, tumor/cancer. N, normal. Kaplan–Meier survival analysis of AML patients with high or low RBM39 expression using the TCGA and GTEx databases. The difference in prognosis was significant according to the log-rank test. RT-qPCR analysis of RBM39 mRNA expression in different AML cell lines. RT-qPCR was used to detect the knockdown efficiency of RBM39 in AMKL cell lines. Flow cytometry analysis of Annexin V + cells in the shNC and shRBM39 groups. Flow cytometry analysis of apoptotic cells in the shNC and shRBM39 groups. The error bars denote the SD. P values were determined via Mann–Whitney U test and are indicated as *P < 0.05, ***P < 0.001, and ****P < 0.0001. "ns" signifies not significant. "N" refers to biological replicates. Each experiment was performed with three technical replicates. [file 13578_2025_1380_MOESM2_ESM.pdf]

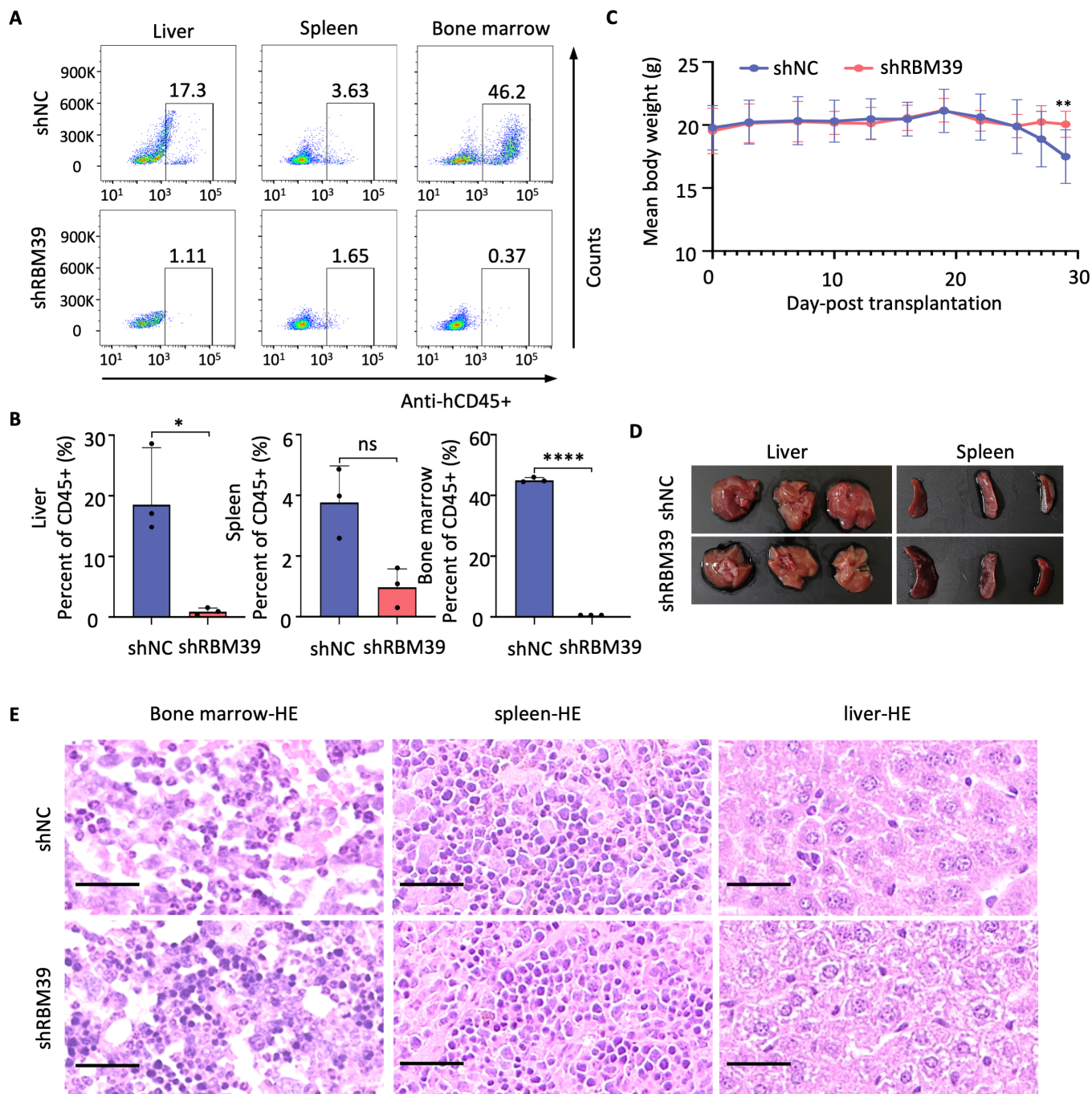

Supplement: Supplementary file 3 — Supplementary Material 3: Fig. S3. Knockdown of RBM39 led to a decreased leukemic burden in the AMKL mouse model. The leukemia burdenin the liver, spleen, and bone marrow was detected by flow cytometry. Percentages of human CD45 + cells in the liver, spleen, and bone marrow. The mean body weights of shNC- and shRBM39-treated AMKL mice. The appearance of the liver and spleen in shNC and shRBM39 AMKL mice. HE staining of bone marrow, spleen, and liver tissue sections. The error bars denote the SD. P values were determined via Mann–Whitney U test and are indicated as *P < 0.05, ***P < 0.001, and ****P < 0.0001. "ns" signifies not significant. Each experiment was performed with three technical replicates. [file 13578_2025_1380_MOESM3_ESM.pdf]

**A**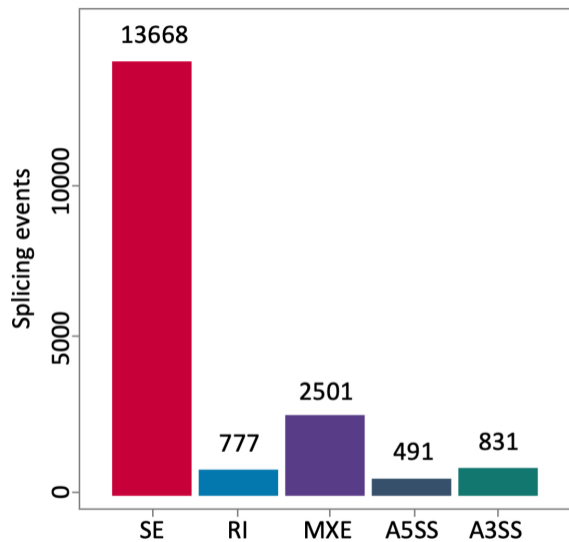**B**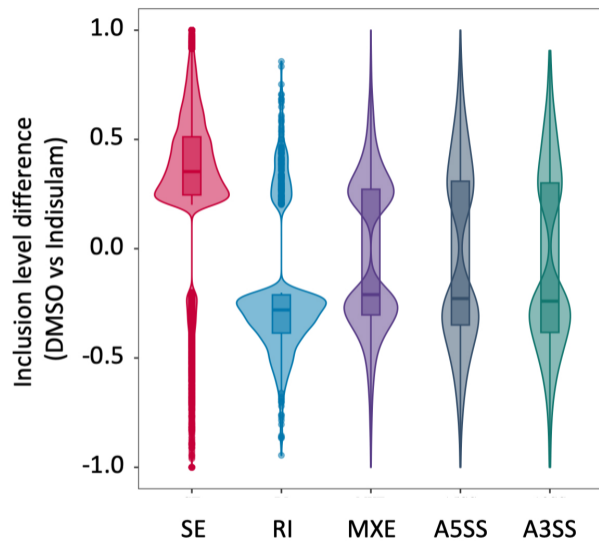

Supplement: Supplementary file 4 — Supplementary Material 4: Fig. S4. RBM39 deletion results in altered RNA splicing. The number of AS events in CMK cells treated with 5 µM indisulam or VC for 24 h. Violin plot of the difference in the inclusion level of AS in CMK cells treated with 5 µM indisulam compared with those treated with VC. [file 13578_2025_1380_MOESM4_ESM.pdf]

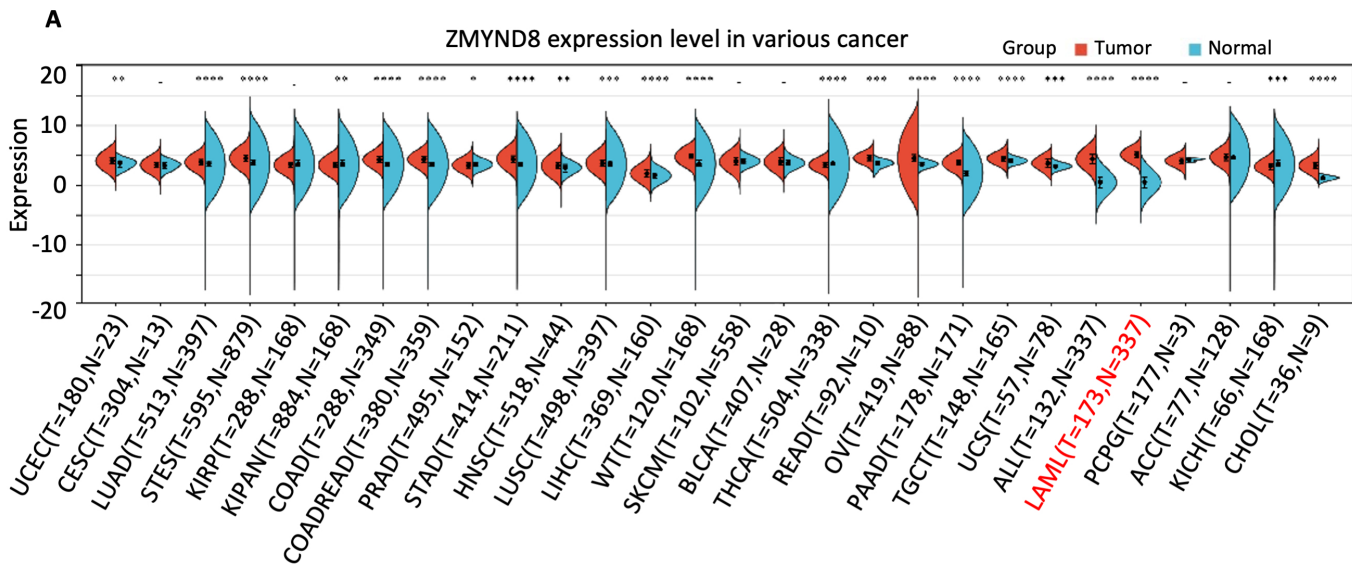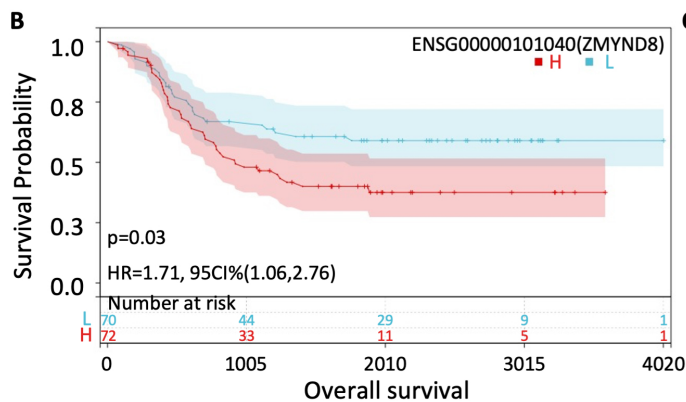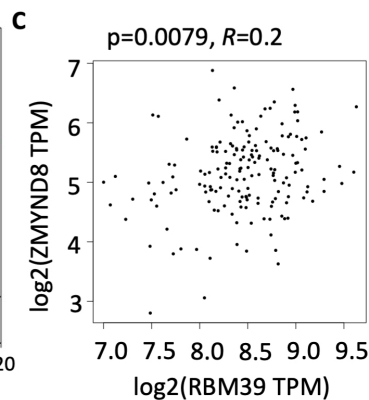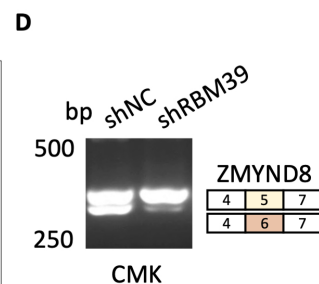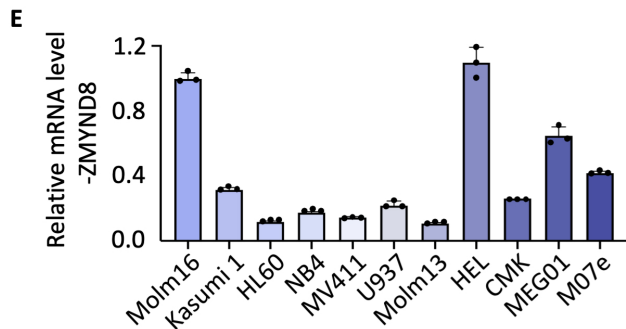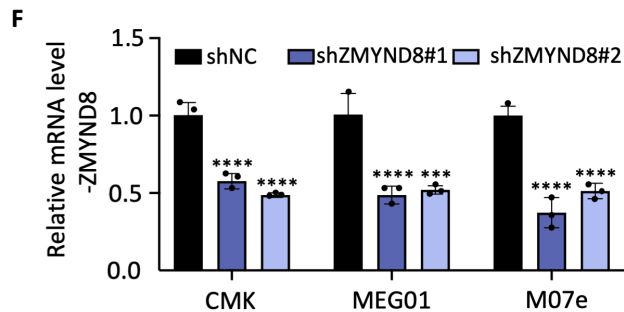

Supplement: Supplementary file 6 — Supplementary Material 6: Fig. S6. ZMYND8 is highly expressed in AMKL and is associated with poor outcomes. ZMYND8 expression is higher in AML samples than in normal samples. Kaplan–Meier survival analysis of AML patients with high or low ZMYND8 expression. The difference was significant according to the Log-Rank test. Pearson correlation analysis between RBM39 and ZMYND8 mRNA expression in AML patients in the TCGA database. TPM, transcripts per million. PCR analysis of the MXE of ZMYND8 in shNC and shRBM39 CMK cells. RT-qPCR analysis of the relative expression level of ZMYND8 in different AML cell lines. RT-qPCR analysis of the knockdown efficiency of ZMYND8 in AMKL cell lines. The error bars denote the SD. P values were determined via Mann–Whitney U test and are indicated as *P < 0.05, **P < 0.01, and ****P < 0.0001. "ns" signifies not significant. Each experiment was performed with three technical replicates. [file 13578_2025_1380_MOESM6_ESM.pdf]

**A**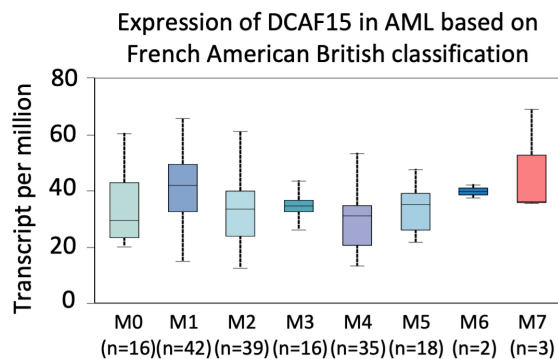**B**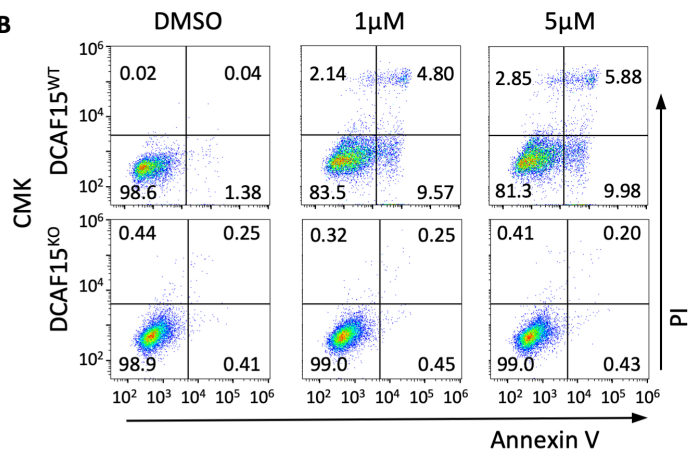**C**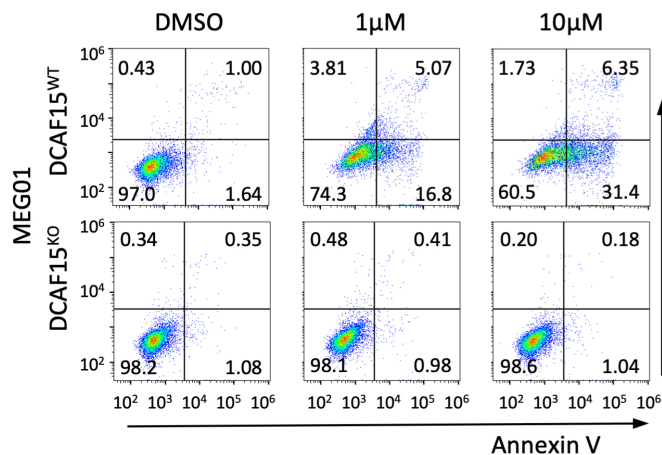**D**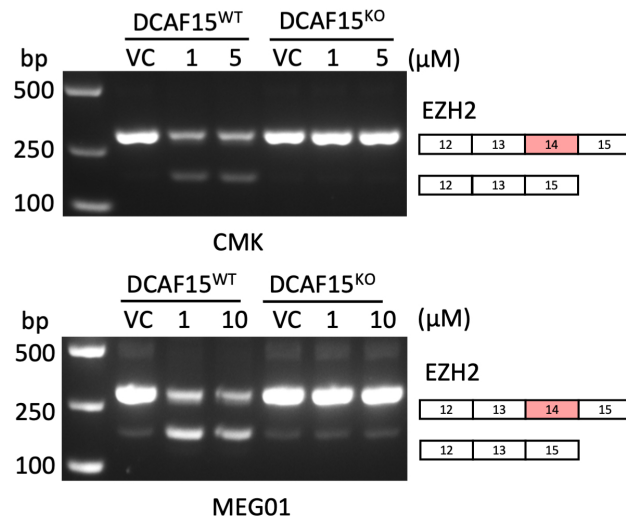**E**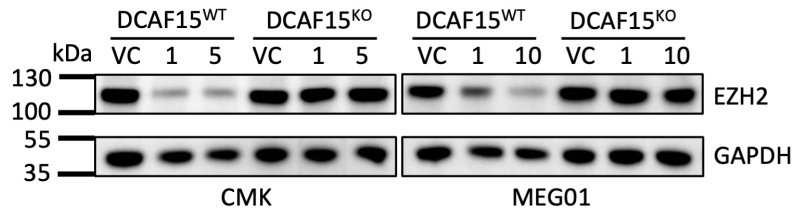

Supplement: Supplementary file 7 — Supplementary Material 7: Fig. S7. DCAF15 is highly expressed in AMKL and is required for the anti-AMKL effect of indisulam. The TCGA database shows the expression of DCAF15 in AML patients based on FAB classification. Assessment of apoptosis via flow cytometry. PCR analysis of the SE of EZH2 in CMK and MEG01cells. Western blot analysis of the EZH2 protein in CMK and MEG01 cells following indisulam treatment. The flow cytometry experiments were performed with three technical replicates. Each experiment was performed with three technical replicates. [file 13578_2025_1380_MOESM7_ESM.pdf]

**A**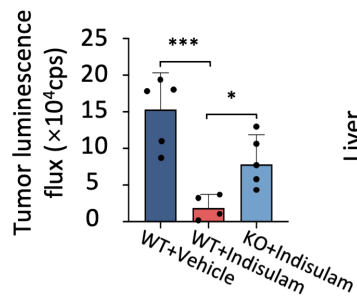**C**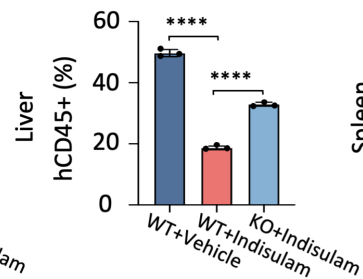**D**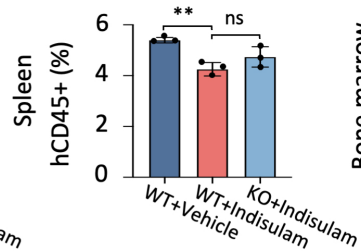**E**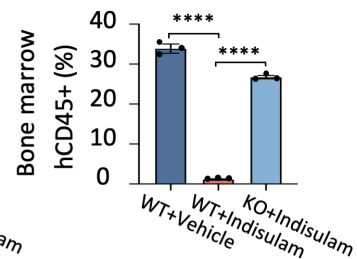**B**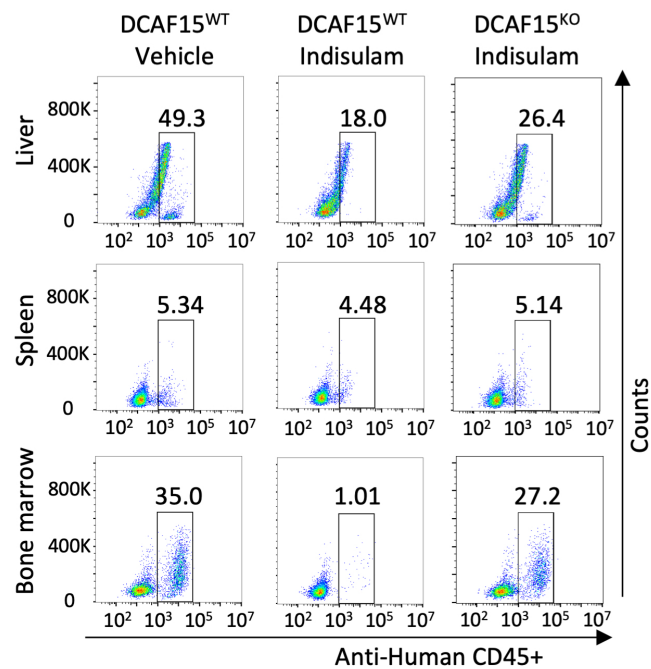**F**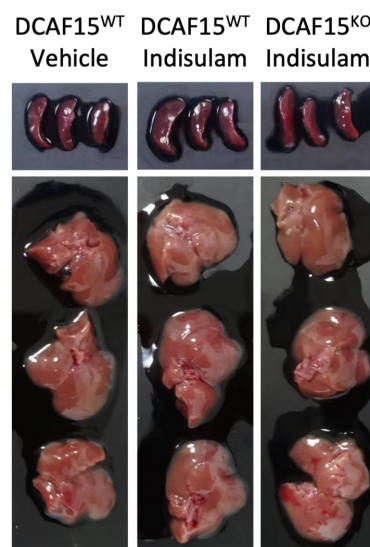

Supplement: Supplementary file 8 — Supplementary Material 8: Fig. S8. The efficacy of indisulam was dependent on DCAF15 in a xenograft mouse model. The tumor fluorescence signal strength of the DCAF15WT + Vehicle, DCAF15WT + indisulam, and DCAF15KO + indisulam groups. Flow cytometry was used to detect human CD45 + cells in the liver, spleen, and bone marrow of the DCAF15WT + Vehicle, DCAF15WT + indisulam, and DCAF15KO + indisulam groups. The percentages of human CD45 + cells in the liver, spleen, and bone marrow in the three groups. The appearance of the spleen and liver in the three groups. The error bars denote the SD. P values were determined via Mann–Whitney U test and are indicated as *P < 0.05, ***P < 0.001, and ****P < 0.0001. "ns" signifies not significant. Each experiment was performed with three technical replicates. [file 13578_2025_1380_MOESM8_ESM.pdf]
