# Supplementary material for: The RBM39 degrader indisulam inhibits acute megakaryoblastic leukemia by altering the alternative splicing of ZMYND8
Source: Cell Biosci. 2025 Apr 13;15:46. doi: 10.1186/s13578-025-01380-3 (PMC11995665; doi:10.1186/s13578-025-01380-3)
Supplement: Supplementary file 5 — Supplementary Material 5: Fig. S5. Indisulam treatment resulted in aberrant RNA splicing and protein changes. Violin plot of differences in protein level changes for genes with different numbers of AS types. Sashimi plots showing the aberrant RNA splicing of ASAH1, ECI2, SELENBP1, and ZMYND8.mRNA expression levels of ASAH1, ECI2, SELENBP1, and ZMYND8 in AML FAB subtypes from the TCGA database. [file 13578_2025_1380_MOESM5_ESM.pdf]

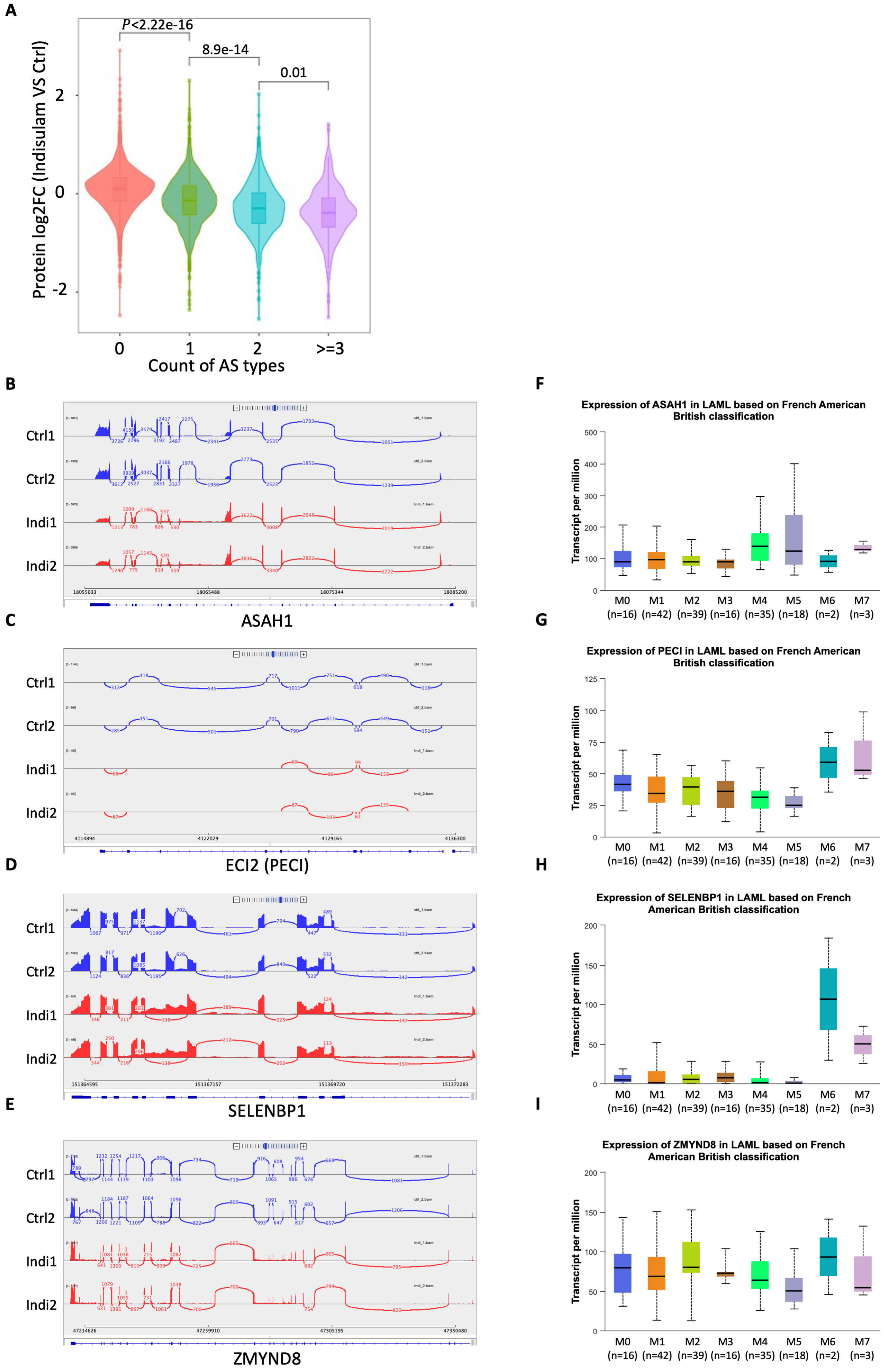
A
P<2.22e-16
8.9e-14
0.01
Protein log2FC (Indisulam VS Ctrl)
2
0
-2
0
1
2
>=3
Count of AS types
B
Ctrl1
Ctrl2
Indi1
Indi2
18055633
18065488
18075344
18085200
ASAH1
C
Ctrl1
Ctrl2
Indi1
Indi2
4114894
4122029
4129165
4136300
ECI2 (PECI)
D
Ctrl1
Ctrl2
Indi1
Indi2
151364595
151367157
151369720
151372283
SELENBP1
E
Ctrl1
Ctrl2
Indi1
Indi2
47214626
47259910
47305195
47350480
ZMYND8
F
Expression of ASAH1 in LAML based on French American British classification
Transcript per million
M0 (n=16)
M1 (n=42)
M2 (n=39)
M3 (n=16)
M4 (n=35)
M5 (n=18)
M6 (n=2)
M7 (n=3)
G
Expression of PECI in LAML based on French American British classification
Transcript per million
M0 (n=16)
M1 (n=42)
M2 (n=39)
M3 (n=16)
M4 (n=35)
M5 (n=18)
M6 (n=2)
M7 (n=3)
H
Expression of SELENBP1 in LAML based on French American British classification
Transcript per million
M0 (n=16)
M1 (n=42)
M2 (n=39)
M3 (n=16)
M4 (n=35)
M5 (n=18)
M6 (n=2)
M7 (n=3)
I
Expression of ZMYND8 in LAML based on French American British classification
Transcript per million
M0 (n=16)
M1 (n=42)
M2 (n=39)
M3 (n=16)
M4 (n=35)
M5 (n=18)
M6 (n=2)
M7 (n=3)
