# Supplementary material for: The RBM39 degrader indisulam inhibits acute megakaryoblastic leukemia by altering the alternative splicing of ZMYND8
Source: Cell Biosci. 2025 Apr 13;15:46. doi: 10.1186/s13578-025-01380-3 (PMC11995665; doi:10.1186/s13578-025-01380-3)
Supplement: Supplementary file 9 — Supplementary Material 9 [file 13578_2025_1380_MOESM9_ESM.pdf]

1. STR identification of MEG01 cell lines.

GENETIC TESTING BIOTECHNOLOGY

Cell Line Authentication Service
STR Profile Report

Sample Submitted By: Dr. Sudan He
Suzhou Institute of Systems Medicine
Email Address: hesudan2018@163.com
Sales Order: 220622C
Cell Line Designation: MEG-01
Date Sample Received: Jun 22th, 2022
Report Date: Jun 23th, 2022

Methodology: Nineteen short tandem repeat (STR) loci plus the gender determining locus, Amelogenin, were amplified using the commercially available EX20 Kit from AGCU. The cell line sample was processed using the ABI Prism® 3130 XL Genetic Analyzer. Data were analyzed using GeneMapper® ID v3.2 software (Applied Biosystems). Appropriate positive and negative controls were run and confirmed for each sample submitted.

Data Interpretation: Cell lines were authenticated using Short Tandem Repeat (STR) analysis as described in 2012 in ANSI Standard (ASN-0002) by the ATCC Standards Development Organization (SDO) and in Capes-Davis et al., Match criteria for human cell line authentication: Where do we draw the line? Int J Cancer. 2013;132(11):2510-9.

GTB™ performs STR Profiling following ISO 9001:2008 and ISO/IEC 17025:2005 quality standards. There are no warranties with respect to the services or results supplied, express or implied, including, without limitation, any implied warranty of merchantability or fitness for a particular purpose. Genetic Testing Biotechnology (GTB) is not liable for any damages or injuries resulting from receipt and/or improper, inappropriate, negligent or other wrongful use of the test results supplied, and/or from misidentification, misrepresentation, or lack of accuracy of those results. Your exclusive remedy against GTB and those supplying materials used in the services for any losses or damage of any kind whatsoever, whether in contract, tort, or otherwise, shall be, at GTB's option, refund of the fee paid for such service or repeat of the service.

NOTE: According to the recommendations of *IJC* on cell line authentication, the report is valid for 3 years since the issue date.

Technical Questions?
GTB Technical Support
+86-512-67486171
service@jsdna.org
Section 505, Yixin BLD
SIP, Suzhou, 215123
Jiangsu, P.R. China

Ordering Questions?
order@jsdna.org
GTB Corporation
+86-512-62806339
Section 303, Yixin BLD
SIP, Suzhou, 215123
Jiangsu, P.R. China

For Research Use ONLY Page 1 of 3 Ver. 3.1.2

GENETIC TESTING BIOTECHNOLOGY

Cell Line Authentication Service
STR Profile Report

Sales Order: 220622C

	Test Results for Submitted Sample			DSMZ Reference Database Profile	
Loci	Query Profile: MEG-01			Database Profile: MEG-01	
Amelogenin	X	Y		X	Y
D3S1358	15				
D13S317	8			8	
D7S820	11			11	
D16S539	9			9	
Penta E	15				
TPOX	8	11		8	11
TH01	7			7	
D2S1338	19				
CSF1PO	10			10	
Penta D	11	13			
D19S433	14	15.2			
vWA	16			16	
D21S11	29				
D18S51	18	22			
D6S1043	14	15	18		
D8S1179	14	15			
D5S818	13			13	
D12S391	19				
FGA	26				

The allele match algorithm compares the 8 core loci plus amelogenin only, even though alleles from all loci will be reported when available.

Note: Loci highlighted in grey (8 core STR loci plus Amelogenin) can be made public to verify cell identity. In order to protect the identity of the donor, ***please do not publish*** *the allele calls from all the STR loci tested. The sample match is based on the reference data available at the time of comparison.*

Explanation of Test Results
Cell lines with ≥80% match are considered to be related; i.e., derived from a common ancestry. Cell lines with between a 55% to 80% match require further profiling for authentication of relatedness.

- ☐ The submitted sample profile is human, but not a match for any profile in the DSMZ STR database.
- ☑ The submitted profile is an exact match for the following human cell line(s) in the DSMZ STR database (8 core loci plus Amelogenin): MEG-01
- ☐ The submitted profile is similar to the following DSMZ human cell line(s):

e-Signature Technician: Digitally signed by Xuekun Chen
DN: cn=Xuekun Chen, o=Genetic Testing Biotechnology (Suzhou), ou=DNA Typing Section, email=order@jsdna.org, c=CN
Date: 2022.06.23 16:37:35 +08'00'

e-Signature Reviewer: Digitally signed by Xiankun Zhao
DN: cn=Xiankun Zhao, o=Genetic Testing Biotechnology (Suzhou), ou=Supervision Section, email=service@jsdna.org, c=CN
Date: 2022.06.23 16:38:08 +08'00'

Addendum: Electropherogram for the customer's sample set 1 of 1

For Research Use ONLY Page 2 of 3 Ver. 3.1.2

GENETIC TESTING BIOTECHNOLOGY

Cell Line Authentication Service
STR Profile Report

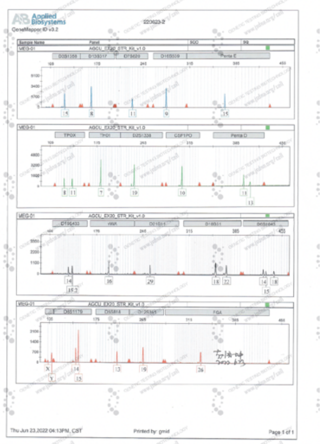

For Research Use ONLY Page 3 of 3 Ver. 3.1.2

2. STR identification of CMK cell lines.

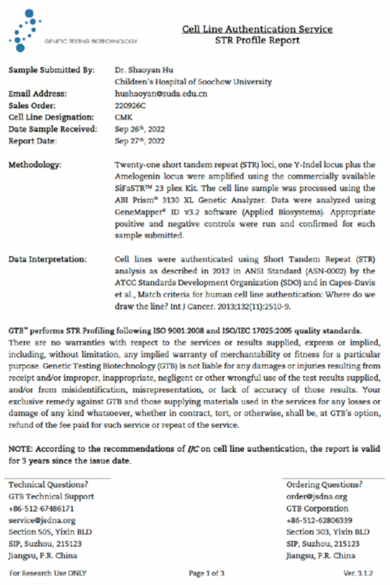

GENETIC TESTING BIOTECHNOLOGY

Cell Line Authentication Service
STR Profile Report

Sample Submitted By: Dr. Shaoyan Hu
Children's Hospital of Soochow University
Email Address: hushaoyan@suda.edu.cn
Sales Order: 220926C
Cell Line Designation: CMK
Date Sample Received: Sep 26th, 2022
Report Date: Sep 27th, 2022

Methodology: Twenty-one short tandem repeat (STR) loci, one Y-Indel locus plus the Amelogenin locus were amplified using the commercially available SiFaSTR™ 23 plex Kit. The cell line sample was processed using the ABI Prism® 3130 XL Genetic Analyzer. Data were analyzed using GeneMapper® ID v3.2 software (Applied Biosystems). Appropriate positive and negative controls were run and confirmed for each sample submitted.

Data Interpretation: Cell lines were authenticated using Short Tandem Repeat (STR) analysis as described in 2012 in ANSI Standard (ASN-0002) by the ATCC Standards Development Organization (SDO) and in Capes-Davis et al., Match criteria for human cell line authentication: Where do we draw the line? Int J Cancer. 2013;132(11):2510-9.

GTB™ performs STR Profiling following ISO 9001:2008 and ISO/IEC 17025:2005 quality standards. There are no warranties with respect to the services or results supplied, express or implied, including, without limitation, any implied warranty of merchantability or fitness for a particular purpose. Genetic Testing Biotechnology (GTB) is not liable for any damages or injuries resulting from receipt and/or improper, inappropriate, negligent or other wrongful use of the test results supplied, and/or from misidentification, misrepresentation, or lack of accuracy of those results. Your exclusive remedy against GTB and those supplying materials used in the services for any losses or damage of any kind whatsoever, whether in contract, tort, or otherwise, shall be, at GTB's option, refund of the fee paid for such service or repeat of the service.

NOTE: According to the recommendations of *IJC* on cell line authentication, the report is valid for 3 years since the issue date.

Technical Questions?
GTB Technical Support
+86-512-67486171
service@jsdna.org
Section 505, Yixin BLD
SIP, Suzhou, 215123
Jiangsu, P.R. China

Ordering Questions?
order@jsdna.org
GTB Corporation
+86-512-62806339
Section 303, Yixin BLD
SIP, Suzhou, 215123
Jiangsu, P.R. China

For Research Use ONLY | Page 1 of 3 | Ver. 3.1.2

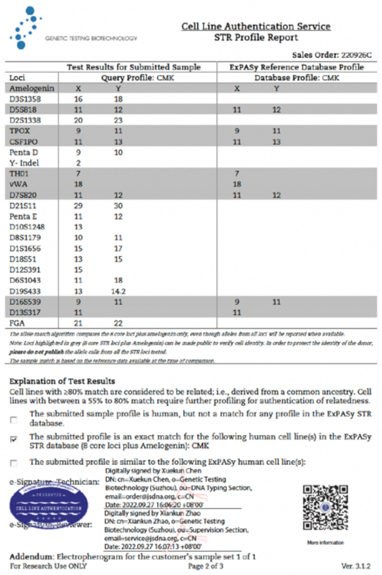

GENETIC TESTING BIOTECHNOLOGY

Cell Line Authentication Service
STR Profile Report

Sales Order: 220926C

	Test Results for Submitted Sample		ExPASy Reference Database Profile	
Loci	Query Profile: CMK		Database Profile: CMK	
Amelogenin	X	Y	X	Y
D3S1358	16	18		
D5S818	11	12	11	12
D2S1338	20	23		
TPOX	9	11	9	11
CSF1PO	11	13	11	13
Penta D	9	10		
Y- Indel	2			
TH01	7		7	
vWA	18		18	
D7S820	11	12	11	12
D21S11	29	30		
Penta E	11	12		
D10S1248	13			
D8S1179	10	11		
D1S1656	15	17		
D18S51	13	15		
D12S391	15			
D6S1043	11	18		
D19S433	13	14.2		
D16S539	9	11	9	11
D13S317	11		11	
FGA	21	22		

The allele match algorithm compares the 8 core loci plus amelogenin only, even though alleles from all loci will be reported when available.
Note: Loci highlighted in grey (8 core STR loci plus Amelogenin) can be made public to verify cell identity. In order to protect the identity of the donor, ***please do not publish*** *the allele calls from all the STR loci tested.*
The sample match is based on the reference data available at the time of comparison.

Explanation of Test Results
Cell lines with ≥80% match are considered to be related; i.e., derived from a common ancestry. Cell lines with between a 55% to 80% match require further profiling for authentication of relatedness.

☐ The submitted sample profile is human, but not a match for any profile in the ExPASy STR database.
☑ The submitted profile is an exact match for the following human cell line(s) in the ExPASy STR database (8 core loci plus Amelogenin): CMK
☐ The submitted profile is similar to the following ExPASy human cell line(s):

e-Signature Technician: Digitally signed by Xuekun Chen
DN: cn=Xuekun Chen, o=Genetic Testing Biotechnology (Suzhou), ou=DNA Typing Section, email=order@jsdna.org, c=CN
Date: 2022.09.27 16:06:20 +08'00'

e-Signature Reviewer: Digitally signed by Xiankun Zhao
DN: cn=Xiankun Zhao, o=Genetic Testing Biotechnology (Suzhou), ou=Supervision Section, email=service@jsdna.org, c=CN
Date: 2022.09.27 16:07:13 +08'00'

More information

Addendum: Electropherogram for the customer's sample set 1 of 1

For Research Use ONLY | Page 2 of 3 | Ver. 3.1.2

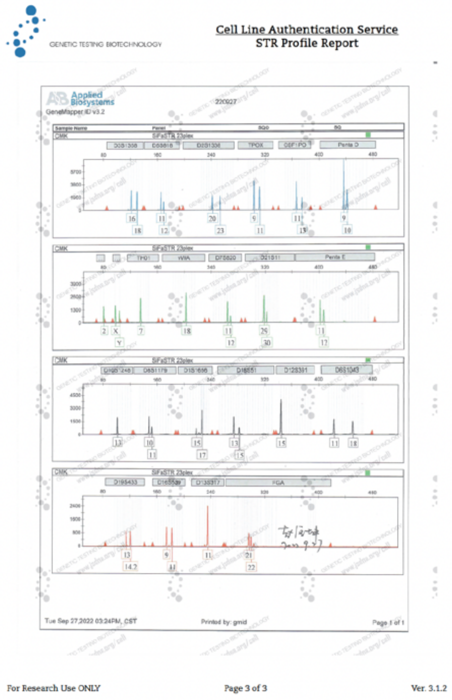

3. STR identification of UT-7 cell lines.

GENETIC TESTING BIOTECHNOLOGY

Cell Line Authentication Service
STR Profile Report

Sample Submitted By: Dr. Jian Pan
Children's Hospital of Soochow University
Email Address: panjian2008@163.com
Sales Order: 231127D
Cell Line Designation: UT-7
Date Sample Received: Nov 27th, 2023
Report Date: Nov 28th, 2023

Methodology: Twenty-one short tandem repeat (STR) loci plus the Amelogenin locus were amplified using the commercially available SiFaSTR™ 23 plex Kit. The cell line sample was processed using the ABI Prism® 3130 XL Genetic Analyzer. Data were analyzed using GeneMapper® ID v3.2 software (Applied Biosystems). Appropriate positive and negative controls were run and confirmed for each sample submitted.

Data Interpretation: Cell lines were authenticated using Short Tandem Repeat (STR) analysis as described in 2021 in ANSI Standard (ASN-0002) by the ATCC Standards Development Organization (SDO) and in Jamie L. Almeida et al., Authentication of Human and Mouse Cell Lines by Short Tandem Repeat (STR) DNA Genotype Analysis. Assay Guidance Manual. PMID: 23805434. Bookshelf ID: NBK144066.

GTB™ performs STR Profiling following ISO 9001:2008 and ISO/IEC 17025:2005 quality standards.
There are no warranties with respect to the services or results supplied, express or implied, including, without limitation, any implied warranty of merchantability or fitness for a particular purpose. Genetic Testing Biotechnology (GTB) is not liable for any damages or injuries resulting from receipt and/or improper, inappropriate, negligent or other wrongful use of the test results supplied, and/or from misidentification, misrepresentation, or lack of accuracy of those results. Your exclusive remedy against GTB and those supplying materials used in the services for any losses or damage of any kind whatsoever, whether in contract, tort, or otherwise, shall be, at GTB's option, refund of the fee paid for such service or repeat of the service.

NOTE: According to the recommendations of *IJC* on cell line authentication, the report is valid for 3 years since the issue date.

Technical Questions?
GTB Technical Support
+86-512-67486171
service@jsdna.org
Section 505, Yixin BLD
SIP, Suzhou, 215123
Jiangsu, P.R. China

Ordering Questions?
order@jsdna.org
GTB Corporation
+86-512-62806339
Section 303, Yixin BLD
SIP, Suzhou, 215123
Jiangsu, P.R. China

For Research Use ONLY Page 1 of 3 Ver. 3.1.2

GENETIC TESTING BIOTECHNOLOGY

Cell Line Authentication Service
STR Profile Report

Sales Order: 231127D

	Test Results for Submitted Sample			ExPASy Reference Database Profile		
Loci	Query Profile: UT-7			Database Profile: UT-7		
Amelogenin	X	Y				
D3S1358	16			16		
D5S818	12			12		
D2S1338	18	23				
TPOX	10			10		
CSF1PO	12			12		
Penta D	9	11				
TH01	6	9		6	9	
vWA	14	18	19	14	18	19
D7S820	8			8		
D21S11	31.2			31.2		
Penta E	16	17				
D10S1248	14	16				
D8S1179	11	15		11	15	
D1S1656	16					
D18S51	14	17		14	17	
D12S391	18	19				
D6S1043	10	19				
D19S433	14	14.2				
D16S539	10	12		10	12	
D13S317	8			8		
FGA	23			23		

The allele match algorithm compares the 13 core STR loci only, even though alleles from all loci will be reported when available.
Note: Loci highlighted in grey (13 core STR loci) can be made public to verify cell identity. In order to protect the identity of the donor, ***please do not publish*** *the allele calls from all the STR loci tested.*
The sample match is based on the reference data available at the time of comparison.

Explanation of Test Results

Cell lines with ≥80% match are derived from the same donor. Cell lines with between a 70% to 79% match require further profiling for authentication of relatedness. Cell lines with <70% match are very unlikely to be from the same donor.

- ☐ The submitted sample profile is human, but not a match for any profile in the ExPASy STR database.
- ☑ The submitted profile is an exact match for the following human cell line(s) in the ExPASy STR database (13 core STR loci): UT-7
- ☐ The submitted profile is similar to the following ExPASy human cell line(s):

e-Signature, Technician: Digitally signed by Xuekun Chen
DN: cn=Xuekun Chen, o=Genetic Testing Biotechnology (Suzhou), ou=DNA Typing Section, email=order@jsdna.org, c=CN
Date: 2023.11.28 17:51:30 +08'00'

e-Signature, Reviewer: Digitally signed by Xiankun Zhao
DN: cn=Xiankun Zhao, o=Genetic Testing Biotechnology (Suzhou), ou=Supervision Section, email=service@jsdna.org, c=CN
Date: 2023.11.28 17:52:05 +08'00'

More information

Addendum: Electropherogram for the customer's sample set 1 of 1

For Research Use ONLY Page 2 of 3 Ver. 3.1.2

GENETIC TESTING BIOTECHNOLOGY

Cell Line Authentication Service
STR Profile Report

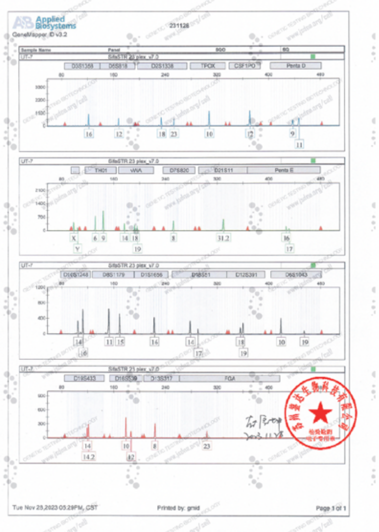

For Research Use ONLY Page 3 of 3 Ver. 3.1.2

4. STR identification of M07e cell lines.

GENETIC TESTING BIOTECHNOLOGY

Cell Line Authentication Service
STR Profile Report

Sample Submitted By: Dr. Jian Pan
Children's Hospital of Soochow University
Email Address: panjian2008@163.com
Sales Order: 231219B
Cell Line Designation: M-07e-New
Date Sample Received: Dec 19th, 2023
Report Date: Dec 19th, 2023

Methodology: Twenty-one short tandem repeat (STR) loci plus the Amelogenin locus were amplified using the commercially available SiFaSTR™ 23 plex Kit. The cell line sample was processed using the ABI Prism® 3130 XL Genetic Analyzer. Data were analyzed using GeneMapper® ID v3.2 software (Applied Biosystems). Appropriate positive and negative controls were run and confirmed for each sample submitted.

Data Interpretation: Cell lines were authenticated using Short Tandem Repeat (STR) analysis as described in 2021 in ANSI Standard (ASN-0002) by the ATCC Standards Development Organization (SDO) and in Jamie L. Almeida et al., Authentication of Human and Mouse Cell Lines by Short Tandem Repeat (STR) DNA Genotype Analysis. Assay Guidance Manual. PMID: 23805434. Bookshelf ID: NBK144066.

GTB™ performs STR Profiling following ISO 9001:2008 and ISO/IEC 17025:2005 quality standards.
There are no warranties with respect to the services or results supplied, express or implied, including, without limitation, any implied warranty of merchantability or fitness for a particular purpose. Genetic Testing Biotechnology (GTB) is not liable for any damages or injuries resulting from receipt and/or improper, inappropriate, negligent or other wrongful use of the test results supplied, and/or from misidentification, misrepresentation, or lack of accuracy of those results. Your exclusive remedy against GTB and those supplying materials used in the services for any losses or damage of any kind whatsoever, whether in contract, tort, or otherwise, shall be, at GTB's option, refund of the fee paid for such service or repeat of the service.

NOTE: According to the recommendations of *IJC* on cell line authentication, the report is valid for 3 years since the issue date.

Technical Questions?
GTB Technical Support
+86-512-67486171
service@jsdna.org
Section 505, Yixin BLD
SIP, Suzhou, 215123
Jiangsu, P.R. China

Ordering Questions?
order@jsdna.org
GTB Corporation
+86-512-62806339
Section 303, Yixin BLD
SIP, Suzhou, 215123
Jiangsu, P.R. China

For Research Use ONLY   Page 1 of 3   Ver. 3.1.2

GENETIC TESTING BIOTECHNOLOGY

Cell Line Authentication Service
STR Profile Report

Sales Order: 231219B

	Test Results for Submitted Sample		ExPASy Reference Database Profile	
Loci	Query Profile: M-07e-New		Database Profile: M-07e	
Amelogenin	X			
D3S1358	16	19	16	19
D5S818	11		11	
D2S1338	19	25		
TPOX	8		8	
CSF1PO	9	10	9	10
Penta D	9			
TH01	6	8	6	8
vWA	16	18	16	18
D7S820	11		11	
D21S11	30	32.2	30	32.2
Penta E	12	14		
D10S1248	14	15		
D8S1179	13	16	13	16
D1S1656	13	16		
D18S51	15	20	15	20
D12S391	23	24		
D6S1043	13	19		
D19S433	14	15		
D16S539	11		11	
D13S317	10	11	10	11
FGA	18	22	18	22

The allele match algorithm compares the 13 core STR loci only, even though alleles from all loci will be reported when available.
*Note: Loci highlighted in grey (13 core STR loci) can be made public to verify cell identity. In order to protect the identity of the donor, **please do not publish** the allele calls from all the STR loci tested.*
The sample match is based on the reference data available at the time of comparison.

Explanation of Test Results
Cell lines with ≥80% match are derived from the same donor. Cell lines with between a 70% to 79% match require further profiling for authentication of relatedness. Cell lines with <70% match are very unlikely to be from the same donor.

☐ The submitted sample profile is human, but not a match for any profile in the ExPASy STR database.
☑ The submitted profile is an exact match for the following human cell line(s) in the ExPASy STR database (13 core STR loci): M-07e
☐ The submitted profile is similar to the following ExPASy human cell line(s):

e-Signature Technician: Digitally signed by Xuekun Chen
DN: cn=Xuekun Chen, o=Genetic Testing Biotechnology (Suzhou), ou=DNA Typing Section, email=order@jsdna.org, c=CN
Date: 2023.12.19 15:14:22 +08'00'

e-Signature Reviewer: Digitally signed by Xiankun Zhao
DN: cn=Xiankun Zhao, o=Genetic Testing Biotechnology (Suzhou), ou=Supervision Section, email=service@jsdna.org, c=CN
Date: 2023.12.19 15:17:10 +08'00'

More information

Addendum: Electropherogram for the customer's sample set 1 of 1

For Research Use ONLY   Page 2 of 3   Ver. 3.1.2

GENETIC TESTING BIOTECHNOLOGY

Cell Line Authentication Service
STR Profile Report

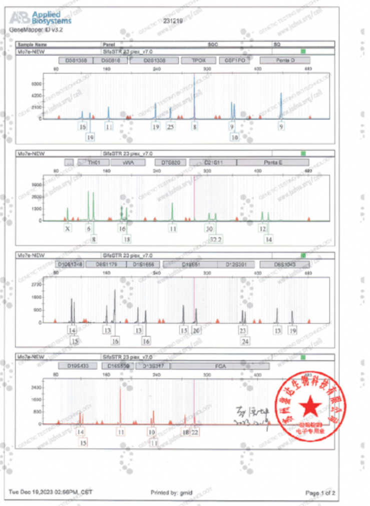

For Research Use ONLY   Page 3 of 3   Ver. 3.1.2

5. STR identification of K-562 cell lines.

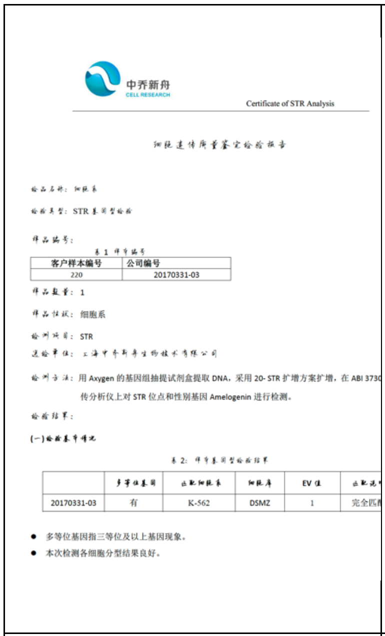

中乔新舟 CELL RESEARCH

Certificate of STR Analysis

细胞遗传质量鉴定检验报告

检品名称：细胞系

检验类型：STR基因型检验

样品编号：

表1 样本编号

客户样本编号	公司编号
220	20170331-03

样品数量：1

样品性状：细胞系

检测项目：STR

送检单位：上海中乔新舟生物技术有限公司

检测方法：用 Axygen 的基因组抽提试剂盒提取 DNA，采用 20- STR 扩增方案扩增，在 ABI 3730

传分析仪上对 STR 位点和性别基因 Amelogenin 进行检测。

检验结果：

(一)检验基本情况

表 2：样本基因型检验结果

	多等位基因	匹配细胞系	细胞库	EV 值	[illegible]
20170331-03	有	K-562	DSMZ	1	完全匹

- 多等位基因指三等位及以上基因现象。
- 本次检测各细胞分型结果良好。

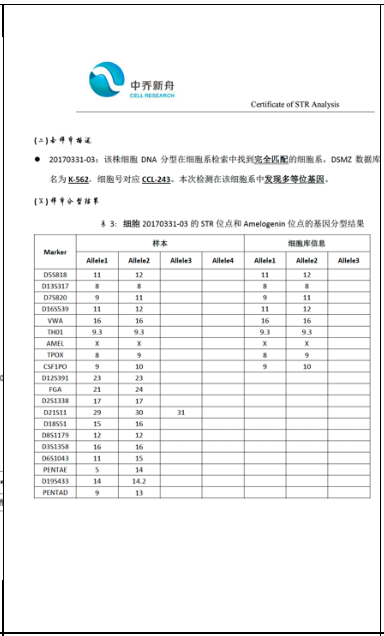

中乔新舟 CELL RESEARCH

Certificate of STR Analysis

(二)各样本描述

- 20170331-03：该株细胞 DNA 分型在细胞系检索中找到完全匹配的细胞系，DSMZ 数据库

名为 K-562，细胞号对应 CCL-243。本次检测在该细胞系中发现多等位基因。

(三)样本分型结果

表 3：细胞 20170331-03 的 STR 位点和 Amelogenin 位点的基因分型结果

Marker	样本				细胞库信息		
	Allele1	Allele2	Allele3	Allele4	Allele1	Allele2	Allele3
D5S818	11	12			11	12	
D13S317	8	8			8	8	
D7S820	9	11			9	11	
D16S539	11	12			11	12	
VWA	16	16			16	16	
TH01	9.3	9.3			9.3	9.3	
AMEL	X	X			X	X	
TPOX	8	9			8	9	
CSF1PO	9	10			9	10	
D12S391	23	23					
FGA	21	24					
D2S1338	17	17					
D21S11	29	30	31				
D18S51	15	16					
D8S1179	12	12					
D3S1358	16	16					
D6S1043	11	15					
PENTAE	5	14					
D19S433	14	14.2					
PENTAD	9	13					

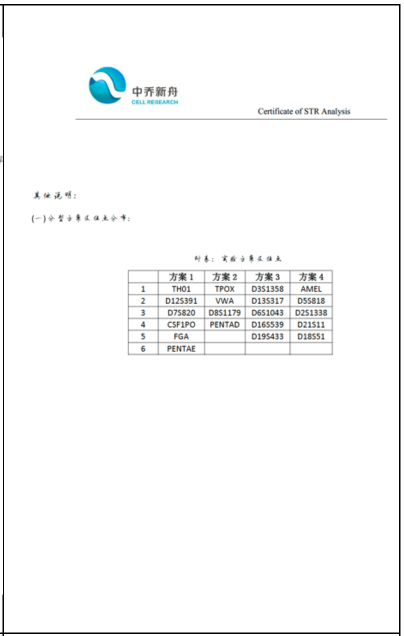

中乔新舟 CELL RESEARCH

Certificate of STR Analysis

其他说明：

(一)分型方案及位点分布：

附表：实验方案及位点

	方案 1	方案 2	方案 3	方案 4
1	TH01	TPOX	D3S1358	AMEL
2	D12S391	VWA	D13S317	D5S818
3	D7S820	D8S1179	D6S1043	D2S1338
4	CSF1PO	PENTAD	D16S539	D21S11
5	FGA		D19S433	D18S51
6	PENTAE			

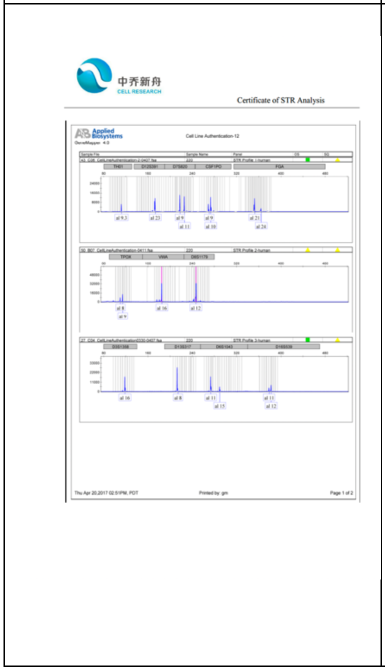

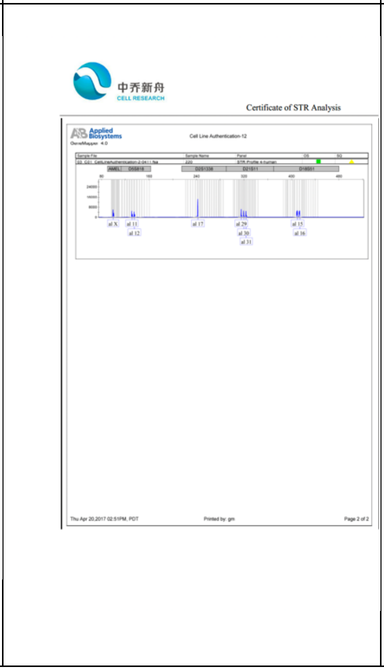

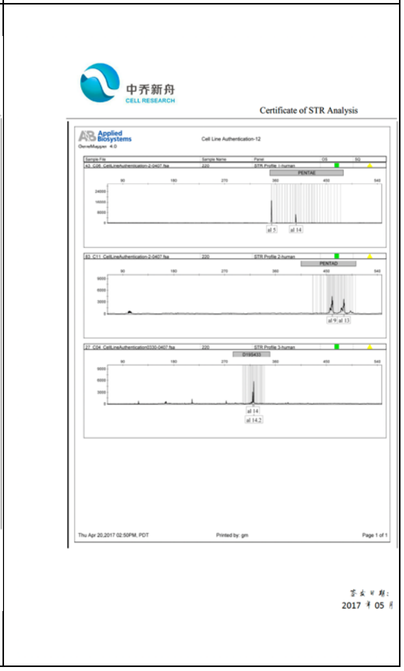

6. STR identification of U937 cell lines.

AZENTA LIFE SCIENCES

Cell Line Authentication Report

AZENTA LIFE SCIENCES

Cell Line Authentication Report

Customer: Zhiheng Li

Quotation Number: 80-1139924657_R2

Completion Date: 03/06/2023

1. **Sample ID: U937**
2. **Original Material: Cell pellet**
3. **Methods:**

1).Genomic DNA was extracted from the cell pellets provided by the customer.

2).Samples, together with positive and negative control were amplified using GenePrint 10 System (Promega).

3). Amplified products were processed using the ABI3730xl Genetic Analyzer.

4). Data were analyzed using GeneMapper4.0 software and then compared with the ATCC, DSMZ, JCRB and RIKEN databases for reference matching.

4. **Results:**

1) 10 Loci STR Profile:

Genetic Site	Cell Bank information		Customer sample	
(Locus)	U-937		U937	
Amelogenin	X	X	X	X
CSF1PO	12	12	12	12
D13S317	10	12	10	12
D16S539	12	12	12	12
D5S818	12	12	12	12
D7S820	9	11	9	11
THO1	6	9.3	6	9.3
TPOX	8	11	8	11
vWA	14	15	14	15
D21S11			27	29
Percent match between the sample and the database profile： 100%				

Summary:

Your cell line is considered to be "identical" to the reference cell line in the Cell Bank STR database, as the STR profile yields a 100% match.

Notes:

AZENTA LIFE SCIENCES

1. P=100% x (2xM)/N; M=18, N=36 P=100% x (2x18)/36=100%

M: number of the matching peaks; N: number of all peaks

2. Based on the ANSI Standard, cell lines with ≥80% match are considered to be related; i.e., derived from a common ancestry. Cell lines with between a 55% to 80% match require further profiling for authentication of relatedness.

3. The short tandem repeat (STR) profile generated by Azenta is indicative only of the sample sent to Azenta at the time it was sent. This data and analysis are for research use only.

AZENTA LIFE SCIENCES

2) Electrophoretogram

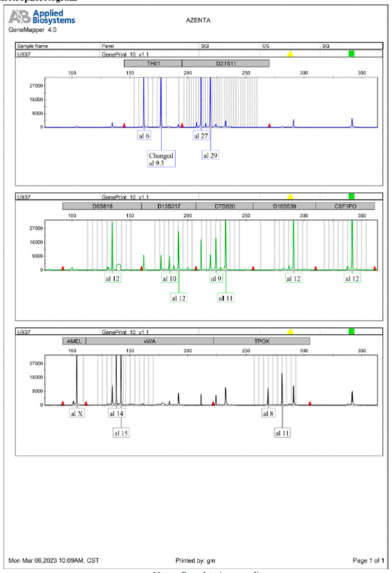

Note：Raw data in appendix
